# PWWP2B Fine‐Tunes Adipose Thermogenesis by Stabilizing HDACs in a NuRD Subcomplex

**DOI:** 10.1002/advs.202102060

**Published:** 2021-06-27

**Authors:** Linyu Yan, Weiwei Jin, Qingwen Zhao, Xuan Cui, Ting Shi, Yingjiang Xu, Feiyan Li, Wenfang Jin, Zhe Zhang, Zhao Zhang, Qi‐Qun Tang, Dongning Pan

**Affiliations:** ^1^ Key Laboratory of Metabolism and Molecular Medicine of the Ministry of Education Department of Biochemistry and Molecular Biology of School of Basic Medical Sciences Fudan University Shanghai 200 032 China

**Keywords:** adipocyte thermogenesis, histone deacetylation, PWWP2B, the NuRD complex

## Abstract

Histone deacetylases (HDACs) are widely involved in many biological processes, as well as in control of brown and beige adipose physiology, but the precise molecular mechanisms by which HDACs are assembled into transcriptional machinery to fine‐tune thermogenic program remain ill‐defined. PWWP domain containing 2b (PWWP2B), which is identified as a component of the nucleosome remodeling and deacetylation complex (NuRD), interacts and stabilizes HDAC1/2 at the thermogenic gene promoters to suppress their expression. Ablation of *Pwwp2b* promotes adipocyte thermogenesis and ameliorates diet‐induced obesity in vivo. Intriguingly, *Pwwp2b* is not only a brown fat‐enriched gene but also dramatically induced by cold and sympathetic stimulation, which may serve as a physiological brake to avoid over‐activation of thermogenesis in brown and beige fat cells.

## Graphical abstract

This work reveals a negative regulation mechanism to fine‐tune adipocyte thermogenesis, and highlights PWWP domain containing 2b (PWWP2B) as an intrinsic brake to keep thermogenic activity appropriately. PWWP2B stabilizes HDAC1/2 in a NuRD subcomplex, and promotes NuRD‐mediated deacetylation on thermogenic gene promoters. PWWP2B‐HDACs coordinate to prevent dissipating too much energy from activation of thermogenic program.

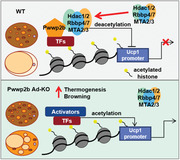

## 1 Introduction

Two types of thermogenic adipocytes, brown and beige fat cells, are now appreciated to play an essential role in systemic metabolism. Thermogenic adipocytes harbor the ability to appropriately activate or repress the thermogenic gene program in response to physiological and environmental stimuli. A set of transcriptional and epigenetic regulators orchestrate adaptive thermogenesis by changing histone marks and chromatin architectures at specific gene promoters.^[^ ^1^ ^]^ Histone acetylation is one of the most abundant epigenetic marks that exerts a profound influence on transcription. The dynamic turnover of acetylation state of a given chromatin locus is managed by histone acetyltransferases (HATs) and histone deacetylases (HDACs). HDACs, which remove acetyl groups from histones or non‐histone substrates, have been highlighted as they are widely involved in tumorigenesis, immune disorder, neurodegenerative diseases, as well as in control of brown and beige adipose physiology.^[^ ^2^ ^]^ It is reported that HDAC1 negatively regulates brown fat‐specific gene activation through coordinated removal of acetylation and deposition of methylation on histone H3 lysine 27 (H3K27).^[^ ^3^ ^]^ Enhanced *Ucp1* expression was also observed in brown and/or beige cells upon HDAC3 inhibitor treatment and in *Hdac3* knockout mice.^[^ ^4^, ^5^, ^6^ ^]^ In accord with these data, Class I HDACs (including HDACs 1, 2, 3, and 8) selective inhibitor, MS275, promotes mitochondrial biogenesis and respiration in primary brown fat cells, increases systemic energy expenditure in both *db/db* mice and diet‐induced obesity mouse models.^[^ ^7^, ^8^ ^]^ Apart from Class I HDACs, HDAC6 and HDAC11 are also reported to be involved in the regulation of adipose thermogenesis.^[^ ^9^, ^10^, ^11^ ^]^ HDACs have therefore emerged as targets for therapeutic interventions for insulin resistance and type 2 diabetes mellitus (T2DM).^[^ ^12^ ^]^

However, the fact that HDACs execute global regulation in a variety of cell types makes them difficult to be useful targets for obesity‐associated diseases due to severe side effects.^[^ ^13^, ^14^, ^15^, ^16^, ^17^ ^]^ HDAC complex may be recruited to specific DNA loci through physically interacting with defined transcription factors or regulators in a cellular context. Transcriptional factor ZEB1 (zinc‐finger E‐box‐binding homeobox 1) is reported to interact with HDAC1 to modulate metastasis in non‐small cell lung cancer.^[^ ^18^ ^]^ ZEB2 cooperates with HDAC complex to control schwann cell differentiation and remyelination.^[^ ^19^ ^]^ ZFPM1 (zinc finger protein, multitype 1; also known as FOG‐1) enrolls HDAC1 to repress GATA‐1 target genes during erythroid cell maturation.^[^ ^20^ ^]^ Bcl6‐dependent follicular T cells development is mediated by a complex formed by Bcl6, Osteopontin, and Mi‐2*β*‐nucleosome‐remodeling deacetylase complex.^[^ ^21^ ^]^ Thus, an alternative strategy to alleviate side effects of HDAC inhibitors is to aim the tissue‐ or cell‐specific component in HDAC protein complexes.^[^ ^17^, ^22^, ^23^ ^]^ In this regard, it will be helpful to unravel how HDACs are assembled into transcriptional machinery to fine‐tune thermogenic gene expression.

In this manuscript, we identified PWWP domain containing 2b (PWWP2B) acting as a scaffold to stabilize HDAC1/2 protein in the nucleosome remodeling and deacetylation (NuRD) subcomplex to modulate expression of thermogenic program in brown and beige adipocytes. *Pwwp2b* is a brown fat‐enriched gene compared to white fat tissues (WAT), and its expression pattern is correlated well with the induction of *Ucp1* in adipose tissues. Unexpectedly, PWWP2B serves as a built‐in brake for adipose thermogenesis by promoting deacetylation on the *Ucp1* promoter. Based on these observations, we propose that PWWP2B bridges the assembly of the NuRD subcomplex in transcription machineries to fine‐tune thermogenic program.

## 2 Results

### 2.1 PWWP2B Protein is Enriched in Brown and Beige Adipose Tissues

**Figure 1:**
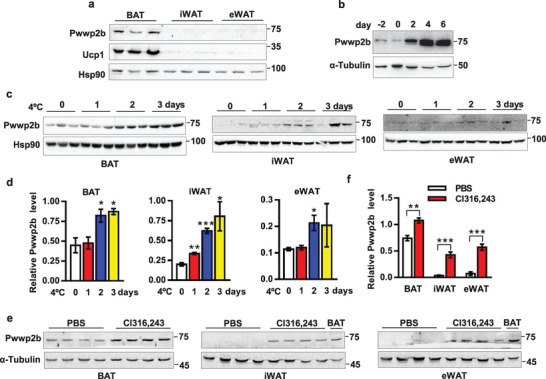
*Pwwp2b* is induced by *β*‐adrenergic signaling in fat tissues. a) PWWP2B protein level in brown and white adipose tissues of C57BL/6 mice. *n* = 3, 12‐week‐old male mice. b) PWWP2B level in differentiating immortalized brown adipocytes. c) PWWP2B level in BAT, iWAT, and eWAT after cold challenge for indicated time. *n* = 3 mice. d) Quantification of PWWP2B level in (c). *n* = 3. *P < 0.05, **P < 0.01, ***P < 0.001 versus the control (0 day) by unpaired two‐tailed Student's *t*‐test. The data shown are mean ± SEM. e) C57BL/6 mice are intraperitoneally (i.p.) injected Cl316243 at a dose of 0.5 ug g^−1^ body weight for 5 days. PWWP2B level in fat tissues is analyzed. *n* = 4 mice. f) Quantification of PWWP2B level in (e). *n* = 4. **P < 0.01, *P < 0.001 by unpaired two‐tailed Student's *t*‐test. The data shown are mean ± SEM.

To understand how brown fat‐enriched molecular components control adaptive thermogenesis, we queried our previously published RNA sequencing (RNA‐seq) (GEO: GSE56367) dataset to search for novel transcriptional or epigenetic regulators that are preferentially expressed in brown adipose tissue (BAT).^[^ ^24^ ^]^ As *Pwwp2b* messenger RNA (mRNA) level in BAT was fivefold compared to that of epididymal WAT (eWAT), *Pwwp2b* was identified as a candidate (Figure S1a,b, Supporting Information). PWWP2B is a functionally uncharacterized protein containing a conserved PWWP (Proline‐Tryptophan‐Tryptophan‐Proline) domain at its C‐terminal, which usually binds nucleosome and recruits their associated chromatin‐modifying enzymes to the target locus on chromatin.^[^ ^25^ ^]^ To first reveal *Pwwp2b* expression distribution in mouse tissues, we performed quantitative PCR (QPCR) analysis to measure *Pwwp2b* mRNA abundance. *Pwwp2b* mRNA is ubiquitously expressed in all tissues examined (Figure S1c, Supporting Information). Consistent with the RNA‐seq data, the levels of *Pwwp2b* protein and mRNA were strikingly higher in BAT compared to in inguinal WAT (iWAT) and eWAT (**Figure** **1**a; Figure S1c, Supporting Information). Whereas *Pwwp2a*, a close homolog of *Pwwp2b*, had no expression preference in brown versus white fat tissues (Figure S1d, Supporting Information). *Pwwp2b* is expressed in both stromal vascular fraction (SVF) and mature adipocytes isolated from BAT (Figure S1e, Supporting Information). Moreover, in immortalized brown fat cell line the PWWP2B level increased progressively during brown adipocyte differentiation and peaked at late differentiation stage (day 4 to day 6) (Figure 1b). The enriched expression of *Pwwp2b* in brown fat tissue and in mature brown fat cells indicates that it may be involved in governing the functional properties of brown adipocytes.

Cold challenge and noradrenergic cascade activate thermogenesis in BAT and recruit beige adipocytes in WAT.^[^ ^26^ ^]^ The expression of *Pwwp2b* transcript was slightly but significantly induced in BAT and eWAT upon mice were subjected to acute cold exposure (Figure S1f, Supporting Information). PWWP2B protein levels were increased in BAT and WAT after cold exposure for 24 or 48 h (Figure 1c,d). Chronic activation of *β*3‐adrenergic receptor by Cl316243 agonist strongly induced PWWP2B protein expression in adipose tissues, especially in white fat tissues (Figure 1e,f), suggesting PWWP2B may be also involved in the process of white adipose browning. In summary, we herein identified *Pwwp2b* as a novel BAT‐enriched component and the expression of *Pwwp2b* is induced in brown and beige adipose tissues by thermogenic activation.

### 2.2 Loss of *Pwwp2b* in Adipose Tissues Enhances Adaptive Thermogenic Response and White Fat Browning

**Figure 2:**
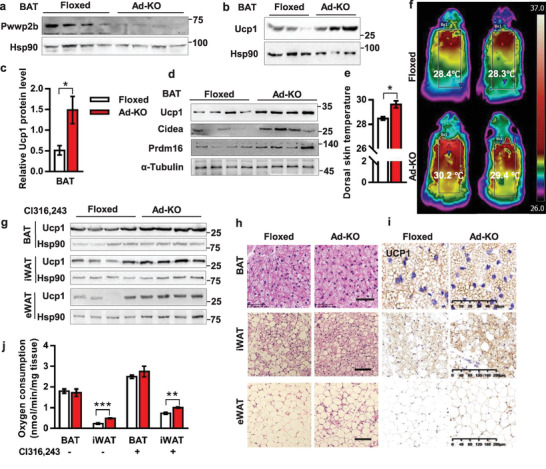
*Pwwp2b* knockout mice demonstrate enhanced thermogenic activity in adipose tissues. a) PWWP2B level in BAT of Floxed and Ad‐KO mice. *n* = 4, 12‐week old male mice. b) UCP1 level in BAT of Floxed and Ad‐KO mice. *n* = 3, 10‐week old male mice. c) Quantification of UCP1 levels in (b). *n* = 3. *P < 0.05 by unpaired two‐tailed Student's *t*‐test. The data shown are mean ± SEM. d) 12‐week‐old mice are housing at 30 ˚C for 2 weeks. Gene expression levels are detected by Western blot. *n* = 4. e) Mice are subjected to 4 ˚C for 7 h. Quantification of dorsal skin temperature measured by an infrared camera. *n* = 4 per group. *P < 0.05 by unpaired two‐tailed Student's *t*‐test. The data shown are mean ± SEM. f) Representative pictures showed dorsal skin temperature of mice in (e). g) Western blot detected UCP1 in fat tissues after CL316243 (0.5 µg g^−1^ body weight) administration by i.p. injection for 7 days (*n* = 4). h) Haematoxylin and eosin (H&E) staining of fat tissues in mice of (g). Scale bar: 50 µm. i) Representative pictures of UCP1 immunostaining of fat tissues in mice of (g). BAT scale bar = 50 µm, iWAT and eWAT scale bar = 200 µm. j) Ex vivo oxygen consumption assay of BAT and iWAT measured by Clark oxygen electrode. Mice are injected CL316243 (0.5 µg g^−1^ body weight) for 7 days (*n* = 4) or without any treatment (*n* = 6). **P < 0.01, ***P < 0.001 by unpaired two‐tailed Student's *t*‐test. The data shown are mean ± SEM.

To study the function of *Pwwp2b* in adipose tissues, we generated adipose tissue‐selective *Pwwp2b* knockout mice (Ad‐KO) by crossing *Pwwp2b^flox/flox^* mice (Floxed) with *Adiponectin‐Cre* mice (Figure S2a, Supporting Information). The *Pwwp2b* knockout mice (*Pwwp2b^flox/flox^* with *Adiponectin‐Cre*) were apparently indistinguishable from the Floxed mice. Then QPCR analysis verified the selective deletion of *Pwwp2b* in BAT and iWAT (Figure S2b, Supporting Information), but no significant reduction in eWAT that may be due to the remaining *Pwwp2b* expression in SVF cells. Reduced PWWP2B protein was observed in BAT (**Figure** **2**a) and fractionated mature brown adipocytes (Figure S2c, Supporting Information) isolated from Ad‐KO mice. *Pwwp2b* Ad‐KO mice consumed similar amount of chow diet and had comparable body weight to Floxed mice when raised at room temperature (23 °C) (Figure S2d,e, Supporting Information). Further gene expression analysis revealed increased mRNA levels of uncoupling protein 1 (*Ucp1*) and PR domain containing 16 (*Prdm16*) in BAT of Ad‐KO mice (Figure S2f, Supporting Information). UCP1 protein level was also upregulated in brown fat tissues of knockout mice (Figure 2b,c). About a twofold increase in *Ucp1*, *Cox8b*, and *Prdm16* mRNA levels were also detected in Ad‐KO iWAT (Figure S2g, Supporting Information). On the other hand, the gene expression screening in eWAT and hematoxylin‐eosin (H&E) staining of brown and white fat tissues did not show much difference between Ad‐KO and Floxed mice (Figure S2h,i, Supporting Information). The adipose *Pwwp2a* mRNA level in knockout mice was similar to that of controls (Figure S2f‐h, Supporting Information).

We next tested the effects of different environmental temperature on BAT adaptive thermogenesis in *Pwwp2b*‐deficient mice. Intriguingly, Ad‐KO mice had more active BAT activities at 30 °C, as revealed by the remarkable upregulation of markers of thermogenesis, including *Ucp1*, *Cidea*, *Prdm16*, *Cox8b* and *Pgc1α* (Figure. 2d; Figure S2j, Supporting Information), implying enhanced diet‐induced BAT thermogenesis upon *Pwwp2b* deletion. Although the *Pwwp2b* knockout mice had similar rectal temperature as Floxed mice when exposed to 4 °C for 6 h (Figure S2k, Supporting Information), elevated dorsal surface temperature was observed in knockout mice (Figure 2e,f). QPCR analysis uncovered increased expression of *Prdm16* and *Atgl* in BAT of knockout mice (Figure S2l, Supporting Information).

Moreover, ablation of *Pwwp2b* sensitized both brown and white adipose tissues to *β*3‐adrenergic receptor agonist Cl316243 administration. Considerable induction of UCP1 protein was seen in BAT, inguinal and epididymal WAT of *Pwwp2b* knockout mice (Figure 2g). Consistent with the gene expression data, Cl316243‐treated knockout mice exhibited more multilocular and positively UCP1‐stained adipocytes in iWAT and eWAT (Figure 2h,i), which are typically associated with the browning process. Elevated oxygen consumption rate (OCR) was also observed in knockout mice iWATs before and after Cl316243 administration (Figure 2j). In contrast, the increase of UCP1 level in BAT of Ad‐KO mice did not elicit the detectable alteration in cell morphology, UCP1 immunostaining, and OCR (Figure 2h–j), possibly due to the alteration was relatively slighter compared to the high basal thermogenic activity in BAT.

### 2.3 *Pwwp2b* Deletion Ameliorates Diet‐Induced Obesity

**Figure 3:**
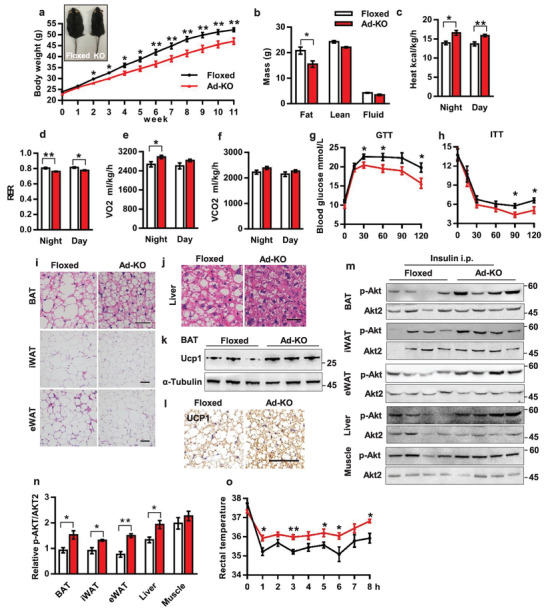
*Pwwp2b* knockout mice gain less body weight on an HFD feeding. a) Body weight of Floxed and knockout mice fed on an HFD. The inset is a representative picture of mice after 11‐week HFD feeding. *n* = 10–11 each genotype, male mice. *P < 0.05, **P < 0.01 versus the control (0 week) by unpaired two‐tailed Student's *t*‐test. The data shown are mean ± SEM. b) Body composition in mice of (a) measured by nuclear magnetic resonance. *n* = 10–11. *P < 0.05 by unpaired two‐tailed Student's *t*‐test. The data shown are mean ± SEM. c–f) Heat production (c), RER (d), oxygen consumption (e), CO_2_ generation (f) measured by metabolic cages for mice in (a). *n* = 10–11. *P < 0.05,**P < 0.01 by unpaired two‐tailed Student's *t*‐test. The data shown are mean ± SEM. g) GTT in mice of (a) after 10‐week HFD feeding. *P < 0.05 versus the control (0 min) by unpaired two‐tailed Student's *t*‐test. The data shown are mean ± SEM. h) ITT in mice of (a) after 11‐week HFD feeding. *P < 0.05 versus the control (0 min) by unpaired two‐tailed Student's *t*‐test. The data shown are mean ± SEM. i) Representative images of H&E stained sections of adipose tissues from mice in (a). Scale bar: 20 µm for BAT, 50 µm for iWAT and eWAT. j) H&E staining of livers in mice of (a). Scale bar: 20 µm. k) UCP1 protein levels in BAT of mice in (a). *n* = 3. l) Representative pictures of BAT UCP1 immunostaining of mice in (a). Scale bar: 50 µm. m) Western blot detected phosphorylated Akt in mice on an HFD for 10 weeks. Mice are sacrificed 15 min after insulin injection. *n* = 4. n) Quantification of the ratio of p‐Akt to total Akt2 in (m). *n* = 4. *P < 0.05, **P < 0.01 by unpaired two‐tailed Student's *t*‐test. The data shown are mean ± SEM. o) Female mice fed on an HFD for 18 weeks are subjected to cold exposure (4 °C). Shown are the core temperatures measured by a rectal probe. *n* = 5 each group. *P < 0.05, **P < 0.01 versus the control (cold 0 h) by unpaired two‐tailed Student's *t*‐test. The data shown are mean±SEM.

To investigate if loss of *Pwwp2b* in adipose tissues alters whole‐body energy expenditure, we then challenged the mice with a high‐fat diet (HFD). The Floxed mice gained weight more rapidly than knockout mice (**Figure** **3**a), although they consumed similar amount of food (Figure S3a, Supporting Information). The decreased body weight of knockout mice was due to less fat mass (Figure 3b), especially significantly decreased amount of inguinal fat tissue deposition (Figure S3b, Supporting Information). An indirect calorimetry study demonstrated that knockout mice had increased heat generation, decreased respiration exchange rate (RER), increased oxygen consumption at night, and unaltered carbon dioxide production (Figure 3c–f). Furthermore, *Pwwp2b*‐knockout mice fed an HFD exhibited moderately but significantly lower blood glucose concentrations after glucose or insulin injection, indicating that genetic disruption of *Pwwp2b* improved glucose tolerance and insulin sensitivity on HFD‐fed mice (Figure 3g,h). Histological analyses demonstrated that knockout mice had markedly reduced size of lipid droplets in BAT, iWAT, and fewer vacuoles in livers (Figure 3i,j).

At molecular levels, increased UCP1 protein, immunostaining, and mRNA levels were shown in BAT of knockout mice (Figure 3k,l; Figure S3c, Supporting Information). The mRNA levels of other thermogenic genes, *Cidea*, *Prdm16*, *Pgc1α*, and/or *Cox8b* were also significantly upregulated in both BAT and iWAT (Figure S3c,d, Supporting Information). The expression of inflammatory marker NF‐*κ*b in iWAT or interleukin 6 (*Il‐6*) in eWAT was decreased in knockout mice (Figure S3d,e, Supporting Information), indicating the ameliorated inflammatory reaction. Improved insulin signaling as assessed by phosphorylated Akt level was displayed in adipose tissues and livers, but not muscles of knockout mice (Figure 3m,n).

The protection of *Pwwp2b* knockout mice from diet‐induced obesity was also observed in female mice (Figure S3f, Supporting Information). The female knockout mice had significantly less fat mass and drastically smaller iWAT and gonadal WAT (gWAT) depots after 18‐week HFD feeding (Figure S3g,h, Supporting Information). Higher core temperature during cold challenge was detected in female knockout mice on an HFD feeding (Figure 3o). Collectively, the enhanced adipose thermogenic gene expression and energy expenditure in *Pwwp2b* knockout mice suggest that PWWP2B serves as a physiologically negative regulator on thermogenesis in both brown and beige adipocytes.

### 2.4 PWWP2B Suppressing Thermogenic Program is Adipocyte‐Autonomous

**Figure 4:**
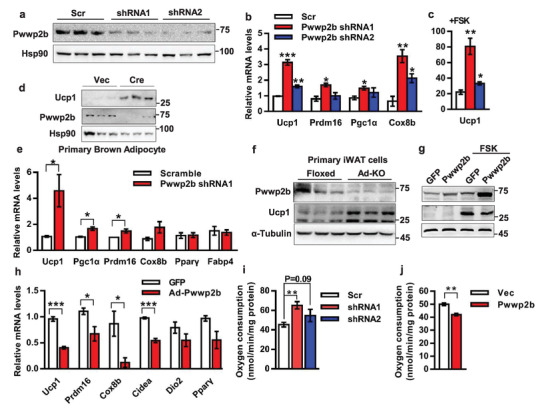
PWWP2B suppresses thermogenic program in a cell‐autonomous manner. a) Lentiviral shRNAs are transduced to immortalized brown preadipocytes and induced differentiation in vitro. PWWP2B level is analyzed by western blot in mature brown adipocytes (day 6). *n* = 3. b) Real‐time QPCR analyzed gene expression in cells of (a). *n* = 3. *P < 0.05, **P < 0.01, ***P < 0.001 versus the scrambled control by unpaired two‐tailed Student's *t*‐test. The data shown are mean ± SEM. c) Cells generated as in (a) are treated by 10 µM FSK for 6 h before the gene expression analysis. *n* = 3. *P < 0.05, **P < 0.01 versus the scrambled control by unpaired two‐tailed Student's *t*‐test. The data shown are mean ± SEM. d) Primary SVF cells are isolated from BAT of *Pwwp2b^flox/flox^* mice. Lentivirus expressing *Cre* infected SVFs to delete *Pwwp2b*. Cells are differentiated to mature adipocytes following western blot analysis. *n* = 3. e) Primary SVF cells isolated from iWAT of C57BL/6 mice are differentiated into beige cells. Lentiviral shRNA infected beige cells on differentiation days 2 and 4, and gene expression analysis is performed on day 6. *n* = 3. *P < 0.05 by unpaired two‐tailed Student's *t*‐test. The data shown are mean ± SEM. f) Primary SVF cells isolated from iWAT of Floxed and knockout mice are differentiated into beige cells. Western blot assay for PWWP2B and UCP1 proteins. *n* = 3. g,h) Adenovirus carrying *Pwwp2b* infected immortalized brown adipocytes on differentiation day 3. Gene expression analysis is performed on day 6 with or without 10µM FSK treatment for 12 h as indicated. h) *n* = 3. *P < 0.05, ***P < 0.001 by unpaired two‐tailed Student's *t*‐test. The data shown are mean ± SEM. i,j) OCR is measured by Clark oxygen electrode in mature adipocytes (day 6) that are knocked down ((i), *n* = 3) or overexpressed *Pwwp2b* ((j,n) = 3). Knockdown or overexpressing lentivirus infected brown preadipocytes following standard induction to mature adipocytes. **P < 0.01 versus the control by unpaired two‐tailed Student's *t*‐test. The data shown are mean ± SEM.

To further explore if the suppressive effects of *Pwwp2b* on thermogenic program are cell‐autonomous, we knocked down *Pwwp2b* expression by lentiviral small hairpin RNAs (shRNA) transduction to immortalized brown preadipocytes. Western blot analysis confirmed the efficiency of the two shRNAs against *Pwwp2b* in mature brown adipocytes (**Figure** **4**a). Decreased *Pwwp2b* level did not disturb the adipogenesis process in brown fat cells, as revealed by general adipogenic marker (*Pparγ*, *Fabp4*, and *Cebpβ*) expression, adipocyte morphology, and oil red O staining (Figure S4a‐c, Supporting Information). The mRNA levels of thermogenic gene *Ucp1*, *Prdm16*, *Pgc1α*, and *Cox8b* were considerably increased after *Pwwp2b* knockdown (Figure 4b). Genes involved in lipolysis and *β*‐oxidation expressed comparable level with the scrambled control cells (Figure S4d, Supporting Information). Reduced *Pwwp2b* expression also promoted brown adipocytes to express higher *Ucp1* level in response to forskolin (FSK), an activator of adenylyl cyclase (Figure 4c). Elevated UCP1 protein level in knockdown cells was further confirmed in primary brown adipocytes isolated from *Pwwp2b^flox/flox^* mice (Figure 4d), where the deletion of *Pwwp2b* was achieved by infecting *Cre* lentivirus to preadipocytes. A similar molecular signature was also observed in primary beige adipocytes when the expression of *Pwwp2b* was curbed by a shRNA (Figure 4e) or in beige cells isolated from *Pwwp2b* knockout and Floxed mice (Figure 4f).

To complement the loss‐of‐function model of *Pwwp2b*, we examined whether increased *Pwwp2b* expression might repress the thermogenic gene program by infecting adenoviral *Pwwp2b* to mature brown adipocytes (Figure 4g) or transducing lentiviral *Pwwp2b* to brown preadipocytes (Figure S4e, Supporting Information). Either strategy could effectively reduce the expression of thermogenic genes (Figure 4h; Figure S4f, Supporting Information). Notably, both knockdown and overexpression of *Pwwp2b* were functionally relevant, as OCR was significantly changed in these cells (Figure 4i,j).

PWWP2B is highly expressed in BAT and induced by thermogenic stimuli, but unexpectedly demonstrates the ability to dampen the activation of thermogenic program. The seemingly paradox observations urge us to find more evidence to support the results. We, therefore, assessed the time course of the induction of UCP1 and PWWP2B in adipose tissues. The increase of UCP1 protein in BAT and iWAT appeared as early as 1 day after Cl316243 administration, whereas PWWP2B expression started to elevate after 2‐day treatment (Figure S5a‐d, Supporting Information). The retarded response of PWWP2B induction to *β*3‐adrenergic agonist coincided with its role as a molecular brake to adipocyte thermogenesis, representing a protective mechanism to avoid hyperactive thermogenesis and maintain energy homeostasis properly.

### 2.5 PWWP2B Stabilizes HDAC1 in a NuRD Subcomplex

**Figure 5:**
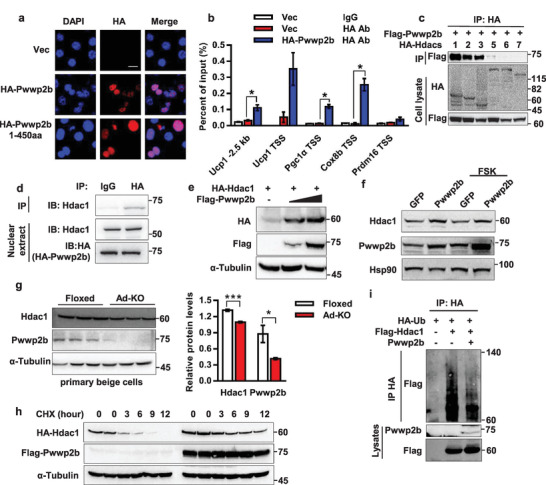
PWWP2B stabilizes HDAC1. a) Immunofluorescence staining shows PWWP2B localizes to mature brown adipocyte nucleus (scale bar: 10µm). Lentiviral *HA‐Pwwp2b* is transduced into brown preadipocytes. Staining is performed on differentiation day 6. b) Representative data of ChIP assay reveal PWWP2B binding on gene promoters. The ChIP assay is performed 3 times. *n* = 3. *P < 0.05 versus data from the vector cells with HA antibody by unpaired two‐tailed Student's *t*‐test. The data shown are mean ± SEM. c) IP is performed when co‐expressing *Pwwp2b* and *Hdacs* plasmids in HEK293T cells, followed by Western blot. d) Brown preadipocytes are infected with HA‐*Pwwp2b* lentivirus, induced differentiation, followed by IP using control IgG or HA antibody. e,f) Presence of PWWP2B increases HDAC1 protein level in e) HEK293T and f) brown adipocytes. The samples used in Figure 5f are the same as in Figure 4g. g) SVF cells isolated from Floxed and knockout mice iWAT are differentiated into beige adipocytes. Western blot showed HDAC1 and PWWP2B levels. Right panel is quantification of HDAC1 and PWWP2B protein levels. *n* = 3. *P < 0.05, ***P < 0.001 by unpaired two‐tailed Student's *t*‐test. The data shown are mean ± SEM. h) CHX chase assay revealed HDAC1's half‐life with and without the presence of PWWP2B in HEK293T cells. i) PWWP2B decreases the ubiquitination of HDAC1 in HEK293T cells. Plasmids are transfected as indicated. 36 h after transfection cells are treated with MG132 for another12 h following IP.

We then wish to elucidate the mechanism by which *Pwwp2b* deficiency activates thermogenic program in adipocytes. Subcellular localization of a protein largely determines its molecular pathways related to the functions. Immunofluorescence staining revealed the PWWP2B protein absolutely existed in brown adipocyte nuclei. Deletion of the PWWP domain on the C‐terminal (PWWP2B 1‐450aa) had no effect on its nucleus localization (**Figure** **5**a). Next, chromatin immunoprecipitation (ChIP) was performed in immortalized brown adipocytes to examine if PWWP2B could bind to thermogenic gene promoters. As illustrated by Figure 5b, PWWP2B was present on the promoter region of *Ucp1*, *Pgc1α*, and *Cox8b*, but not on *Prdm16* transcriptional star site (TSS), implying PWWP2B might affect gene transcription, histone modification, or chromatin architecture by binding to gene promoters.

PWWP2B was previously identified as one of the components in a variant NuRD protein complex in mouse embryonic stem cells (mESCs).^[^ ^27^, ^28^ ^]^ Our liquid chromatography coupled with tandem mass spectrometry (LC‐MS/MS) confirmed HDAC s HDAC1 and HDAC2 co‐existing in the PWWP2B protein immunoprecipitant isolated from mature brown adipocytes (Table S1, Supporting Information). Furthermore, another four elements, including retinoblastoma‐binding protein 4 (RBBP4), RBBP7, metastasis‐associated 1 family member 2 (MTA2) and MTA3, of the NuRD complex were also detected. Co‐immunoprecipitation (IP) assay validated the association between exogenously expressed PWWP2B and HDAC1/2/3 in HEK293T cells, but no interaction could be detected between PWWP2B and members of class II HDACs, HDAC5/6/7 (Figure 5c). Overexpressed PWWP2B at physiological level (see Figure S4e, Supporting Information) also interacted with endogenous HDAC1 in brown adipocytes (Figure 5d). The interaction between PWWP2B and MTA1/2/3 or RBBP4/7 was confirmed by overexpressing tagged plasmids in HEK293T cells (Figure S6a, Supporting Information).

Since HDAC1 is the most abundant protein that can be pulled down by PWWP2B (Figure 5c), we then focused on the interaction between PWWP2B and HDAC1. To assess the region of PWWP2B mediating PWWP2B‐HDAC1 interaction, we co‐transfected plasmids bearing *Hdac1* and full length or truncated *Pwwp2b* to HEK293T cells. A strong interaction was observed with a fragment containing 1–600 (full length), 1–450 or 151–600 amino acids of PWWP2B, and a weaker interaction was shown between PWWP2B 1–150 amino acids and HDAC1 (Figure S6b, Supporting Information). Interestingly, we noticed that the expression of HDAC1 protein was dramatically elevated with the presence of full‐length or truncated PWWP2B, except for PWWP2B 1–150 fragments (Figure S6b, Supporting Information, see cell lysates). Further Western blot analysis confirmed the presence of PWWP2B heightened HDAC1 (Figure 5e) and HDAC2 protein levels (Figure S6c, Supporting Information), but did not increase HDAC5 or RBBP4/7 protein levels. Neither had any effect on HDAC1 mRNA expression (Figure S6d‐f, Supporting Information). PWWP2A also showed the capability to increase HDAC1 protein level when co‐expressed in HEK293T cells (Figure S6g, Supporting Information). However, RBBP4 and RBBP7, the acknowledged partners of HDAC1, did not enhance the expression of HDAC1 protein (Figure S6h, Supporting Information). More importantly, overexpression of PWWP2B in mature brown adipocytes also increased HDAC1 protein level (Figure 5f). Conversely, depletion of *Pwwp2b* in primary beige adipocytes slightly but significantly lowered the HDAC1 protein expression (Figure 5g). The fact that increased HDAC1 protein but not mRNA level by overexpressing PWWP2B led us to predict that the presence of PWWP2B might increase the HDAC1 protein stability. Cycloheximide (CHX) chase analysis and HDAC1 ubiquitination assay showed that PWWP2B stabilized HDAC1 by decreasing its ubiquitination level (Figure 5h,i).

The deubiquitinating enzyme, BRCA1/BRCA2‐containing complex subunit 3 (BRCC3), was present in PWWP2B immunoprecipitant complex (Table S1, Supporting Information) and involved in deubiquitinating HDAC1 and PWWP2B (Figure S7a,b, Supporting Information). BRCC3 interacts and stabilizes HDAC1 or PWWP2B individually (Figure S7c,d, Supporting Information), and the presence of PWWP2B could further augment the BRCC3 interacting with and stabilizing HDAC1 protein (Figure S7c,d, Supporting Information, see the last lane in each Figure). In addition, overexpressed *Brcc3* increased the protein, but not mRNA, levels of PWWP2B and HDAC1 in brown adipocytes (Figure S7e‐g, Supporting Information). A corresponding reduction in the protein levels of PWWP2B and HDAC1 was also observed in brown adipocytes with *Brcc3* knockdown (Figure S7h‐j, Supporting Information). All these data indicate BRCC3 plays a part in stabilizing the PWWP2B‐HDAC1 complex.

### 2.6 PWWP2B Regulates Histone Acetylation Level on the *Ucp1* Promoter

**Figure 6:**
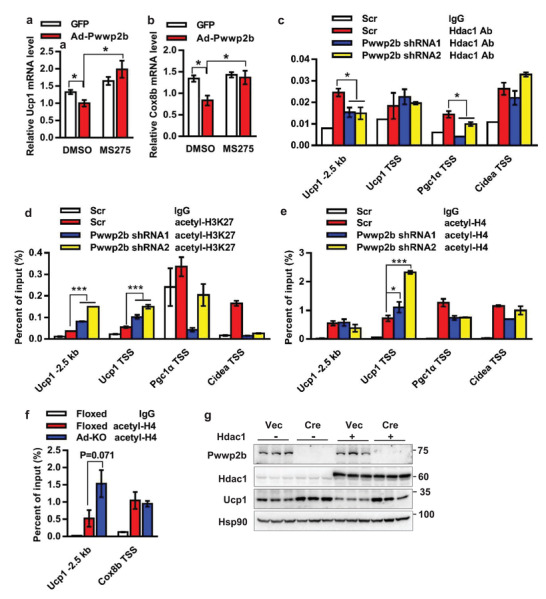
HDAC1 is required for PWWP2B inhibition on thermogenic gene expression. a,b) MS275 abolished PWWP2B suppression effect on a) *Ucp1* and b) *Cox8b* expression. MS275 (2µM) treated the cells for 12 h on day 6. *n* = 3. *P < 0.05 by unpaired two‐tailed Student's *t*‐test. The data shown are mean ± SEM. c) Representative data to show knockdown of *Pwwp2b* reduces HDAC1 association on gene promoters. The ChIP assay is performed in brown adipocytes 3 times. *n* = 3. *P < 0.05 versus data from scrambled cells with Hdac1 antibody by unpaired two‐tailed Student's *t*‐test. The data shown are mean ± SEM. d,e) Representative data to show knockdown of *Pwwp2b* reduces acetylated d) H3K27 and e) H4 levels on *Ucp1* promoter. The ChIP assay is performed in brown adipocytes 3 times. *n* = 3. *P < 0.05, ***P < 0.001 versus data from scrambled cells with d) acetyl‐H3K27 or e) acetyl‐H4 antibody by unpaired two‐tailed Student's *t*‐test. The data shown are mean ± SEM. f) Acetylated H4 level in iWAT of mice administrated Cl316243 for 7 days. *n* = 4. Unpaired two‐tailed Student's *t*‐test. The data shown are mean ± SEM. g) *Pwwp2b* is knocked out in primary brown preadipocytes by lentiviral *Cre* infection. Lentivirus expressing *Hdac1* transduced cells on differentiation day 2 and day 4. Indicated proteins are detected by Western blot.

Histone deacetylation is a well‐defined repressive regulatory mechanism for gene transcription, which could be responsible for the suppressive role of PWWP2B in thermogenesis. In fact, the chemical inhibitor of HDAC1, MS275,^[^ ^7^ ^]^ did abolish PWWP2B‐elicited inhibitory effect on *Ucp1* and *Cox8b* expression (**Figure** **6**a,b). Accordingly, knockdown of *Pwwp2b* decreased HDAC1 binding on *Ucp1* distal and *Pgc1α* TSS regions, implying the abundance of HDAC1 on thermogenic gene promoters at least partly depends on the presence of PWWP2B (Figure 6c). Consequently, the levels of acetylated H3K27 and H4 on *Ucp1* promoter were increased in *Pwwp2b* knockdown cells (Figure 6d,e). In vivo, the increased UCP1 expression in iWAT of knockout mice after chronic Cl316243 treatment (see Figure 2g) was accompanied by the tended elevation of H4 acetylation level at *Ucp1* enhancer region (*p* = 0.071) (Figure 6f). On the other hand, the inhibition effect of HDAC1 on UCP1 expression counted on the presence of PWWP2B. When *Cre* lentivirus depleted *Pwwp2b* in primary brown adipocytes, the HDAC1‐elicited inhibition on UCP1 expression was greatly abolished (Figure 6g). Taken together, we conclude that the functional roles of PWWP2B and HDAC1 in thermogenic adipocytes are interrelated and interdependent.

## 3 Discussion

**Figure 7:**
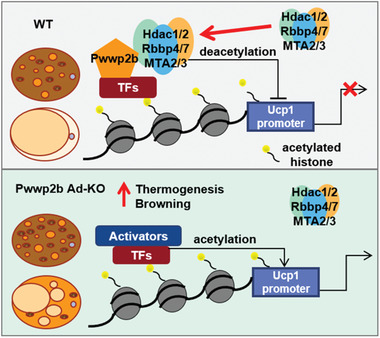
A model of how PWWP2B regulates adipocyte thermogenesis. PWWP2B associates and stabilizes HDAC1/2 in the NuRD subcomplex to promote deacetylation on the thermogenic gene promoters. It thus acts as a molecular brake to restrain hyperactive thermogenesis. In the absence of *Pwwp2b*, less HDAC1/2 sticks to promoters delicately activating the thermogenic program in adipocytes.

In this manuscript, we identified a BAT‐enriched protein PWWP2B curbed adipose thermogenesis by promoting NuRD‐mediated deacetylation on gene promoters (**Figure** **7**). Given the effect of PWWP2B specifically regulating thermogenic gene program, we speculate designated transcriptional factors may be involved in recruitment of PWWP2B to particular chromatin regions. Transcriptional factor ZFP516 that was also detected in the PWWP2B protein complex (Table S1, Supporting Information) was previously reported to activate *Ucp1* promoter and promote browning of iWAT by interacting with PRDM16 and LSD1.^[^ ^29^, ^30^ ^]^ Our preliminary inspection demonstrated that endogenous PWWP2B interacted with overexpressed HA‐ZFP516 in brown adipocytes (Figure S8a, Supporting Information). ZFP516 recruits LSD1 to the promoter regions of BAT‐enriched genes, where it demethylates methylated H3K9 and activates transcription.^[^ ^30^ ^]^ PWWP2B competed against LSD1 to interact with ZFP516 (Figure S8b, Supporting Information). Whereas, PRDM16 facilitated the interaction between PWWP2B and ZFP516 (Figure S8c, Supporting Information), although PRDM16 itself did not interact with PWWP2B (Figure S8d, Supporting Information). ZFP516 knockdown almost completely abrogated PWWP2B binding to thermogenic gene promoters (Figure S8e,f, Supporting Information). Moreover, the existence of PWWP2B promoted ZFP516 interaction with HDAC1 (Figure S8g, Supporting Information), likely due to PWWP2B stabilizes HDAC1 and acts as a scaffold for the assembly of NuRD complex into the ZFP516 transcription apparatus.

One confusing question is the disparity between the expression pattern of *Pwwp2b* and its roles in adipocytes. *Pwwp2b* is a BAT‐enriched gene and induced by cold exposure and *β*‐adrenergic signaling pathway. However, our in vitro and in vivo data show that PWWP2B functions as a negative regulator for thermogenesis through directing and stabilizing HDAC1 on gene promoters. Adaptive thermogenesis‐induced system energy expenditure is deleterious in cancer‐associated cachexia, burn injury, and kidney failure.^[^ ^31^, ^32^, ^33^ ^]^ It is not surprising that intrinsic constraint mechanisms on thermogenesis are developed to avoid caloric waste. PWWP2B is not the only one that acts as a brake on the road of non‐shivering thermogenesis. The cAMP‐CREB axis induced the RNA‐binding protein QKI transcription, but QKI impedes adipose thermogenesis by decreasing *Ucp1* and *Pgc1α* mRNA stability, cytoplasm localization, and translation efficiency.^[^ ^34^ ^]^ The expression level of potassium two pore domain channel subfamily K member 3 (*Kcnk3*) is much higher in brown and beige than in white adipocytes. It is transcriptionally activated by PRDM16 and cold stimuli in adipose tissues. KCNK3‐mediated outward potassium current antagonizes norepinephrine‐induced calcium influx to restrain cAMP production.^[^ ^35^ ^]^ All these “brake” systems are crucial to preserve energy and avoid wasting.

The full length of PWWP2B protein is 600 amino acids, with a PWWP domain at the C‐terminal (498‐583aa). The PWWP domain contains five anti‐parallel *β*‐strands (*β*1‐*β*5), with the Pro‐Trp‐Trp‐Pro motif locating in the *β*2‐strand.^[^ ^36^ ^]^ The PWWP domain interacts with DNA,^[^ ^37^ ^]^ and reads methylated H3K36 and methylated H4K20.^[^ ^38^, ^39^, ^40^, ^41^, ^42^, ^43^ ^]^ PWWP2B, as well as PWWP2A, have been previously reported to be in the deacetylase core of the NuRD complex.^[^ ^27^, ^28^ ^]^ PWWP2A‐MTA complex is enriched at actively transcribed gene bodies,^[^ ^27^, ^28^ ^]^ and recognizes and binds both H2A.Z and H3K36 trimethylation.^[^ ^28^, ^44^ ^]^ In contrast, PWWP2B is enriched on active promoters and enhancers in mESCs.^[^ ^28^ ^]^ PWWP2A and PWWP2B may have redundant and specific functions. Our mass spectrometry results did not identify PWWP2A in PWWP2B immunoprecipitants isolated from mature adipocytes. The PWW2A function in adipocytes and its binding selectivity to distinct chromatin moieties remains to be further explored.

HDAC1 and HDAC2 are incorporated into multiple transcriptional regulatory complexes, including NuRD, SIN3‐HDAC complex, mitotic deacetylase complex (MiDAC), REST corepressor (CoREST) complex, mesoderm induction early response transcriptional regulator (MIER) complex, and RERE‐HDAC complexes, and each has discrete biological functions.^[^ ^45^ ^]^ HDAC inhibitors show promise for the treatment of many diseases, but the limitation is the undesirable side effects due to the diverse functions of HDACs. Designing inhibitors that could target individual HDAC complex or perturbing protein‐protein interactions in complexes is a potential strategy to overcome the side effects. BAT‐enriched PWWP2B stabilizes HDAC1 and HDAC2 in a NuRD subcomplex and suppresses thermogenesis in adipocytes. The insight of functional roles of the PWWP2B‐HDAC1 complex in adaptive thermogenesis may contribute to the novel therapeutics for obesity and the related diseases.

## 4 Experimental Methods

### Animal Studies

All animal studies were performed in accordance with an Institutional Animal Care and Use Committee‐approved protocol of Fudan University. Mice were housed in 12 h light and 12 h dark cycle at 23 °C with free access to water and a standard rodent chow diet unless otherwise specified. *Pwwp2b^flox/flox^* mice were obtained from Beijing Biocytogen Company on C57BL/6 background. To generate adipocyte‐specific *Pwwp2b* knockout mice, *Pwwp2b^flox/flox^* mice were crossed to mice expressing *Cre* recombinase under the control of the adiponectin promoter (Jackson Laboratory, 02 8020). For HFD‐feeding, mice were fed starting at about 6 weeks of age with 60% calories as fat (Research Diets D12492) for the indicated time periods. Glucose tolerance test (GTT) and insulin tolerance test (ITT) were performed in mice after an HFD. Mice fasted for 16 h (GTT) or 5 h (ITT) following intraperitoneal injection with glucose (2g per kg body mass) or insulin (0.75U per kg body mass), respectively. Core body temperature was measured using the rectal electronic thermometer (ALT‐ET03, Shanghai Alcott Biotech CO.LTD). Mice skin temperature was discerned by infrared camera (Flir One, Flir Systems). Body composition of mice was determined after HFD with nuclear magnetic resonance (Minispec LF50, Bruker). For acute cold exposure, mice caged with food withdrawn were placed in a 4 °C environment for indicated time. For thermoneutrality housing, mice were raised at 30 °C for 2 weeks. In each experiment, only the same gender and similar age littermate mice were used as control.

### Indirect Calorimetry

*Pwwp2b* knockout or control mice fed an HFD for 11 weeks (male) or 18 weeks (female) were placed in the Comprehensive Lab Animal Monitoring System (Columbus Instruments) and allowed to acclimate. Mice were monitored for 24 h for oxygen consumption, carbon dioxide production, and heat generation. Data were shown as the average over 12‐h dark and light cycle.

### Oxygen Consumption

Freshly isolated BAT or iWAT depots (about 40 mg) were minced in 1 mL phosphate‐buffered saline supplemented with 25 mM glucose, 1 mM pyruvate, and 2% BSA. Cultured adipocytes were trypsinized and collected by centrifugation. Cell pellets were resuspended in the buffer above. Oxygen consumption was measured with a Clark electrode (Oxygraph^+^ system, Hansatech) and data were normalized to total tissue weight or total protein contents.

### Adipocyte Culture Studies

Immortalized BAT preadipocytes were generated and induced differentiation as described previously.^[^ ^46^ ^]^ Differentiated adipocytes were treated with FSK (10 µM) in some experiments as indicated. Primary brown fat cell culture was set up by isolating brown preadipocytes from *Pwwp2b^flox/flox^* neonatal mice. For differentiation, when SVF cells grew to 70% confluence, cells were incubated in differentiation medium [10% fetal bovine serum DMEM containing 20 nM insulin and 1 nM triiodo‐L‐thyronine (T3)] for 2 days (day ‐2 to day 0). Then cells were exposed to induction medium (DMEM supplemented with 10% fetal bovine serum, 850 nM insulin, 1 nM T3, 500 µM isobutylmethylxanthine, 0.5 µM dexamethasone, and 125 µM indomethacin) for 48 h (day 0 to day 2). The medium was changed to differentiation medium on day 2 and day 4. Mature adipocytes were collected on day 6 for further analysis. To knockout *Pwwp2b* in primary brown fat cells, lentivirus expressing *Cre* recombinase infected SVF cells.

To culture beige adipocytes, SVFs were obtained from iWAT of 15‐day‐old WT and *Pwwp2b* knockout mice. Confluent SVF cells were induced to adipocyte by incubated in induction medium as described above for 48 h (day 0 to day 2). Then cells were maintained in DMEM medium containing 10% FBS, 850 nM insulin, 1nM T3 from Day 2 until day 6. FSK was added to medium for the last 12 h during culture.

### Gene Expression Analysis, Co‐IP, and Mass Spectrometry

The messenger RNA expression level was analyzed by quantitative RT‐PCR. Western blot was used to explore the protein levels. The primer sequences and antibodies used in this study were listed in Table S2 and Table S3, Supporting Information.

For IP assay, HEK293T cell extracts with ectopic expressing tagged plasmids were incubated with anti‐HA beads (Smart lifesciences, SA068001) or anti‐Flag beads (Smart lifesciences, SA042001). Immunoprecipitates were probed with indicated antibodies. To examine the interaction between HDAC1 or ZFP516 with PWWP2B in brown fat cells, lentiviral HA‐PWWP2B or HA‐ZFP516 was transduced to immortalized brown preadipocytes. HA antibody was used to precipitate the HA‐PWWP2B or HA‐ZFP516 protein complex from differentiated mature adipocytes followed by western blot. The HA‐PWW2B protein complex was subjected to LC‐MS/MS analysis (Shanghai Applied Protein Technology Co. Ltd). For the ubiquitination assay, HEK293T cells were treated with 5 µM MG132 for 12 h before harvesting the cells.

### Chromatin IP Assays (ChIP)

Briefly, brown adipocytes overexpressing HA‐*Pwwp2b* or knocking down *Pwwp2b* by lentiviral transduction were fixed in 1% formaldehyde for 5 min at room temperature. Fresh iWAT depots from *Pwwp2b* knockout or WT mice were fixed and ground with dounce homogenizer. Fragmentation of the DNA was achieved by ultrasonic processor (Cole Parmer). The sheared chromatin was then immunoprecipitated with antibodies against HA (Santa Cruz sc‐805X), HDAC1 (Proteintech, 10197‐1‐AP), acetylated H3K27 (Abcam, ab4729), acetylated H4 (Santa Cruz sc‐34263), or rabbit IgG (Sigma). One percent of the chromatin samples were saved as input control. DNA samples from the ChIP were purified with PCR purification kits (Qiagen) and applied to quantification by real‐time PCR. ChIP signal was normalized with input signal.

### Plasmids and Viruses

All the gene open reading frames were amplified by RT‐PCR and verified by sequencing. Human *Hdac* plasmids were kindly provided by Dr. Qunying Lei, Fudan University. Construction and package of knockdown (Addgene, pSP‐108 vector), overexpressing lentivirus (Addgene, 17 398), and adenovirus (Addgene, 16 404) was followed the standard protocol. Lentiviral Cre construct was from addgene (#30 205).

### Virus Transduction and Plasmids Transfection

Lentivirus bearing a shRNA or target genes infected preadipocytes with the supplement of 4 µg mL^−1^ polybrene. Preadipocytes were then selected and grown in culture media with 3 µg mL^−1^ puromycin. Following the standard induction protocol, preadipocytes differentiated to mature adipocytes. Adenovirus infected differentiated adipocytes on day 2 and day 4. The gene expression analysis was usually performed on differentiation day 6. Plasmid transfection to HEK293T cell was carried out following instruction of Lipofectamine 2000 (Thermo Fisher). Western blot or IP assay was performed 48 h after transfection.

### Immunofluorescence Staining

For immunofluorescence staining, brown preadipocytes expressing *HA‐Pwwp2b* were grown on cover slips and induced differentiation in a 24‐well plate. Paraformaldehyde‐fixed cells were incubated with HA antibody (Santa Cruz sc‐805X, 1:100) at 4 °C overnight. Subsequently, Alexa Flour 568‐conjugated secondary antibody (ThermoFisher A‐11004) and DAPI were applied to cells for 60 min. Images were acquired and processed with Confocal Laser Scanning Microscopy (Leica) at the same setting for all the pictures.

### CHX Chase Assay

To determine protein stability of HDAC1 with or without PWWP2B, HEK293T cells expressing *HA‐Hdac1* and *Flag‐Pwwp2b* or vector plasmids were treated by CHX (50 µM) (Meilunbio MB2208) for indicated time. Cell lysates were separated by SDS‐PAGE and HDAC1 protein level was detected by HA antibody (Sigma, F7425).

### Statistical Analysis

All experiments were performed in at least triplicate and the sample size (*n*) as shown in the relevant figure legends. Results were expressed as mean ± standard error of mean (SEM). The statistical difference between two groups was analyzed using unpaired two‐tailed Student's *t*‐test. GraphPad Prism 7.0 software was used for all statistical analysis. The statistical significance was defined as *P* < 0.05, and expressed as *P < 0.05, **P < 0.01, ***P < 0.001.

## Conflict of Interest

The authors declare no conflict of interest.

## Author contributions

L.Y. and W.J. contributed equally to this work. L.Y. and W.J. performed most experiments, acquired data, and analyzed data. Q.Z, X.C., Y.X., T.S., F.L., Z.Z., and W.J. conducted experiments. Z.Z. analyzed RNA‐seq data (GEO: GSE56367). Q.T advised on the manuscript. D.P. designed the project, supervised the study, and drafted the manuscript.

## Supporting information

Supporting Information

## Data Availability

Data sharing is not applicable to this article as no new data were created or analyzed in this study.

## References

[1] T. Inagaki, J. Sakai, S. Kajimura, Nat. Rev. Mol. Cell Biol. 2016, 17, 480. 27251423 10.1038/nrm.2016.62 PMC4956538

[2] K. J. Falkenberg, R. W. Johnstone, Nat. Rev. Drug Discovery 2014, 13, 673. 25131830 10.1038/nrd4360

[3] F. Li, R. Wu, X. Cui, L. Zha, L. Yu, H. Shi, B. Xue, J. Biol. Chem. 2016, 291, 4523. 26733201 10.1074/jbc.M115.677930 PMC4813478

[4] J. Liao, J. Jiang, H. Jun, X. Qiao, M. P. Emont, D. I. Kim, J. Wu, Endocrinology 2018, 159, 2520. 29757434 10.1210/en.2018-00257 PMC6456926

[5] A. Ferrari, R. Longo, E. Fiorino, R. Silva, N. Mitro, G. Cermenati, F. Gilardi, B. Desvergne, A. Andolfo, C. Magagnotti, D. Caruso, E. Fabiani, S. W. Hiebert, M. Crestani, Nat. Commun. 2017, 8, 93. 28733645 10.1038/s41467-017-00182-7 PMC5522415

[6] A. Yuliana, H. F. Jheng, S. Kawarasaki, W. Nomura, H. Takahashi, T. Ara, T. Kawada, T. Goto, Int. J. Mol. Sci. 2018, 19, 2436. 10.3390/ijms19082436 PMC6121552 30126161

[7] A. Galmozzi, N. Mitro, A. Ferrari, E. Gers, F. Gilardi, C. Godio, G. Cermenati, A. Gualerzi, E. Donetti, D. Rotili, S. Valente, U. Guerrini, D. Caruso, A. Mai, E. Saez, E. De Fabiani, M. Crestani, Diabetes 2013, 62, 732. 23069623 10.2337/db12-0548 PMC3581211

[8] A. Ferrari, E. Fiorino, R. Longo, S. Barilla, N. Mitro, G. Cermenati, M. Giudici, D. Caruso, A. Mai, U. Guerrini, E. De Fabiani, M. Crestani, Int. J. Obes. 2017, 41, 289. 10.1038/ijo.2016.191 27795551

[9] S. Jung, M. Han, S. Korm, S. I. Lee, S. Noh, S. Phorl, R. Naskar, K. S. Lee, G. H. Kim, Y. J. Choi, J. Y. Lee, Biochem. Biophys. Res. Commun. 2018, 503, 285. 29890133 10.1016/j.bbrc.2018.06.016

[10] L. Sun, D. E. C. Marin, K. Bian, A. Achille, E. Telles, H. Pei, E. Seto, EBioMedicine 2018, 33, 157. 29958910 10.1016/j.ebiom.2018.06.025 PMC6085537

[11] R. A. Bagchi, B. S. Ferguson, M. S. Stratton, T. Hu, M. A. Cavasin, L. Sun, Y. H. Lin, D. Liu, P. Londono, K. Song, M. F. Pino, L. M. Sparks, S. R. Smith, P. E. Scherer, S. Collins, E. Seto, T. A. McKinsey, JCI Insight 2018, 3, e120159. 10.1172/jci.insight.120159 PMC6129125 30089714

[12] S. Sharma, R. Taliyan, Pharmacol. Res. 2016, 113, 320. 27620069 10.1016/j.phrs.2016.09.009

[13] Y. Li, E. Seto, Cold Spring Harbor Perspect. Med. 2016, 6, a026831. 10.1101/cshperspect.a026831 PMC5046688 27599530

[14] R. Rahhal, E. Seto, Nucleic Acids Res. 2019, 47, 4911. 31162605 10.1093/nar/gkz292 PMC6547430

[15] P. Misztak, P. Panczyszyn‐Trzewik, M. Sowa‐Kucma, Pharmacol. Rep. 2018, 70, 398. 29456074 10.1016/j.pharep.2017.08.001

[16] D. Zhang, X. Hu, R. H. Henning, B. J. Brundel, Cardiovasc. Res. 2016, 109, 519. 26645980 10.1093/cvr/cvv265

[17] R. D. Marcum, I. Radhakrishnan, FEBS Lett. 2020, 594, 2322. 32391601 10.1002/1873-3468.13811 PMC8224932

[18] R. Manshouri, E. Coyaud, S. T. Kundu, D. H. Peng, S. A. Stratton, K. Alton, R. Bajaj, J. J. Fradette, R. Minelli, M. D. Peoples, A. Carugo, F. Chen, C. Bristow, J. J. Kovacs, M. C. Barton, T. Heffernan, C. J. Creighton, B. Raught, D. L. Gibbons, Nat. Commun. 2019, 10, 5125. 31719531 10.1038/s41467-019-12832-z PMC6851102

[19] L. M. Wu, J. Wang, A. Conidi, C. Zhao, H. Wang, Z. Ford, L. Zhang, C. Zweier, B. G. Ayee, P. Maurel, A. Zwijsen, J. R. Chan, M. P. Jankowski, D. Huylebroeck, Q. R. Lu, Nat. Neurosci. 2016, 19, 1060. 27294509 10.1038/nn.4322 PMC4961522

[20] W. Hong, M. Nakazawa, Y. Y. Chen, R. Kori, C. R. Vakoc, C. Rakowski, G. A. Blobel, EMBO J. 2005, 24, 2367. 15920470 10.1038/sj.emboj.7600703 PMC1173144

[21] E. Shen, Q. Wang, H. Rabe, W. Liu, H. Cantor, J. W. Leavenworth, Proc. Natl. Acad. Sci. USA 2018, 115, 6780. 29891681 10.1073/pnas.1805239115 PMC6042103

[22] I. Darlyuk‐Saadon, K. Weidenfeld‐Baranboim, K. K. Yokoyama, T. Hai, A. Aronheim, Biochim. Biophys. Acta 2012, 1819, 1142. 22989952 10.1016/j.bbagrm.2012.09.005 PMC3551276

[23] D. Y. Lee, C. I. Lee, T. E. Lin, S. H. Lim, J. Zhou, Y. C. Tseng, S. Chien, J. J. Chiu, Proc Natl Acad Sci U. S. A. 2012, 109, 1967. 22308472 10.1073/pnas.1121214109 PMC3277521

[24] D. Pan, L. Huang, L. J. Zhu, T. Zou, J. Ou, W. Zhou, Y. X. Wang, Dev. Cell 2015, 35, 568. 26625958 10.1016/j.devcel.2015.11.002 PMC4679478

[25] R. J. Gilbert, S. M. Dalla, C. J. Froelich, M. I. Wallace, G. Anderluh, Trends Biochem. Sci. 2014, 39, 510. 25440714 10.1016/j.tibs.2014.09.002

[26] W. Wang, P. Seale, Nat. Rev. Mol. Cell Biol. 2016, 17, 691. 27552974 10.1038/nrm.2016.96 PMC5627770

[27] T. Zhang, G. Wei, C. J. Millard, R. Fischer, R. Konietzny, B. M. Kessler, J. W. R. Schwabe, N. Brockdorff, Nat. Commun. 2018, 9, 3798. 30228260 10.1038/s41467-018-06235-9 PMC6143588

[28] S. Link, R. Spitzer, M. Sana, M. Torrado, M. C. Volker‐Albert, E. C. Keilhauer, T. Burgold, S. Punzeler, J. Low, I. Lindstrom, A. Nist, C. Regnard, T. Stiewe, B. Hendrich, A. Imhof, M. Mann, J. P. Mackay, M. Bartkuhn, S. B. Hake, Nat. Commun. 2018, 9, 4300. 30327463 10.1038/s41467-018-06665-5 PMC6191444

[29] J. Dempersmier, A. Sambeat, O. Gulyaeva, S. M. Paul, C. S. Hudak, H. F. Raposo, H. Y. Kwan, C. Kang, R. H. Wong, H. S. Sul, Mol. Cell 2015, 57, 235. 25578880 10.1016/j.molcel.2014.12.005 PMC4304950

[30] A. Sambeat, O. Gulyaeva, J. Dempersmier, K. M. Tharp, A. Stahl, S. M. Paul, H. S. Sul, Cell Rep. 2016, 15, 2536. 27264172 10.1016/j.celrep.2016.05.019 PMC4916264

[31] M. Petruzzelli, M. Schweiger, R. Schreiber, R. Campos‐Olivas, M. Tsoli, J. Allen, M. Swarbrick, S. Rose‐John, M. Rincon, G. Robertson, R. Zechner, E. F. Wagner, Cell Metab. 2014, 20, 433. 25043816 10.1016/j.cmet.2014.06.011

[32] D. Patsouris, P. Qi, A. Abdullahi, M. Stanojcic, P. Chen, A. Parousis, S. Amini‐Nik, M. G. Jeschke, Cell Rep. 2015, 13, 1538. 26586436 10.1016/j.celrep.2015.10.028 PMC4662886

[33] S. Kir, H. Komaba, A. P. Garcia, K. P. Economopoulos, W. Liu, B. Lanske, R. A. Hodin, B. M. Spiegelman, Cell Metab. 2016, 23, 315. 26669699 10.1016/j.cmet.2015.11.003 PMC4749423

[34] H. Lu, Z. Ye, Y. Zhai, L. Wang, Y. Liu, J. Wang, W. Zhang, W. Luo, Z. Lu, J. Chen, EMBO Rep. 2020, 21, e47929. 31868295 10.15252/embr.201947929 PMC6944952

[35] Y. Chen, X. Zeng, X. Huang, S. Serag, C. J. Woolf, B. M. Spiegelman, Cell 2017, 171, 836. 28988768 10.1016/j.cell.2017.09.015 PMC5679747

[36] S. Qin, J. Min, Trends Biochem. Sci. 2014, 39, 536. 25277115 10.1016/j.tibs.2014.09.001

[37] G. B. Rona, E. Eleutherio, A. S. Pinheiro, Biophys. Rev. 2016, 8, 63. 28510146 10.1007/s12551-015-0190-6 PMC5425739

[38] A. Vezzoli, N. Bonadies, M. D. Allen, S. M. Freund, C. M. Santiveri, B. T. Kvinlaug, B. J. Huntly, B. Gottgens, M. Bycroft, Nat. Struct. Mol. Biol. 2010, 17, 617. 20400950 10.1038/nsmb.1797

[39] A. Dhayalan, A. Rajavelu, P. Rathert, R. Tamas, R. Z. Jurkowska, S. Ragozin, A. Jeltsch, J. Biol. Chem. 2010, 285, 26114. 20547484 10.1074/jbc.M109.089433 PMC2924014

[40] S. M. Sankaran, A. W. Wilkinson, J. E. Elias, O. Gozani, J. Biol. Chem. 2016, 291, 8465. 26912663 10.1074/jbc.M116.720748 PMC4861420

[41] R. Guo, L. Zheng, J. W. Park, R. Lv, H. Chen, F. Jiao, W. Xu, S. Mu, H. Wen, J. Qiu, Z. Wang, P. Yang, F. Wu, J. Hui, X. Fu, X. Shi, Y. G. Shi, Y. Xing, F. Lan, Y. Shi, Mol. Cell 2014, 56, 298. 25263594 10.1016/j.molcel.2014.08.022 PMC4363072

[42] H. Wen, Y. Li, Y. Xi, S. Jiang, S. Stratton, D. Peng, K. Tanaka, Y. Ren, Z. Xia, J. Wu, B. Li, M. C. Barton, W. Li, H. Li, X. Shi, Nature 2014, 508, 263. 24590075 10.1038/nature13045 PMC4142212

[43] Y. Wang, B. Reddy, J. Thompson, H. Wang, K. Noma, J. R. Yates, S. Jia, Mol. Cell 2009, 33, 428. 19250904 10.1016/j.molcel.2009.02.002 PMC2673476

[44] S. Punzeler, S. Link, G. Wagner, E. C. Keilhauer, N. Kronbeck, R. M. Spitzer, S. Leidescher, Y. Markaki, E. Mentele, C. Regnard, K. Schneider, D. Takahashi, M. Kusakabe, C. Vardabasso, L. M. Zink, T. Straub, E. Bernstein, M. Harata, H. Leonhardt, M. Mann, R. A. Rupp, S. B. Hake, EMBO J. 2017, 36, 2263. 28645917 10.15252/embj.201695757 PMC5538766

[45] C. J. Millard, P. J. Watson, L. Fairall, J. Schwabe, Trends Pharmacol. Sci. 2017, 38, 363. 28139258 10.1016/j.tips.2016.12.006

[46] D. Pan, M. Fujimoto, A. Lopes, Y. X. Wang, Cell 2009, 137, 73. 19345188 10.1016/j.cell.2009.01.051 PMC2688451

