# Supplementary material for: PWWP2B Fine‐Tunes Adipose Thermogenesis by Stabilizing HDACs in a NuRD Subcomplex
Source: Adv Sci (Weinh). 2021 Jun 27;8(16):2102060. doi: 10.1002/advs.202102060 (PMC8373154; doi:10.1002/advs.202102060)
Supplement: Supplementary file 1 — Supporting Information [file ADVS-8-2102060-s001.pdf]

© 2021 Wiley-VCH GmbH

ADVANCED SCIENCE
Open Access

Supporting Information

for *Adv. Sci.*, DOI: 10.1002/advs.202102060

PWWP2B Fine-Tunes Adipose Thermogenesis by Stabilizing HDACs in a NuRD Subcomplex

*Linyu Yan, Weiwei Jin, Qingwen Zhao, Xuan Cui, Ting Shi, Yingjiang Xu, Feiyan Li, Wenfang Jin, Zhe Zhang, Zhao Zhang, Qi-Qun Tang, and Dongning Pan**

Supplementary Information for

PWWP2B Fine-tunes Adipose Thermogenesis by Stabilizing HDACs in a NuRD Subcomplex

*Linyu Yan[#], Weiwei Jin[#], Qingwen Zhao, Xuan Cui, Ting Shi, Yingjiang Xu, Feiyan Li, Wenfang Jin, Zhe Zhang, Zhao Zhang, Qi-Qun Tang, Dongning Pan**

Key Laboratory of Metabolism and Molecular Medicine of the Ministry of Education
Department of Biochemistry and Molecular Biology of School of Basic Medical Sciences
Fudan University
Shanghai 200032, China

Corresponding author:
Dongning Pan, Ph.D.
E-mail: dongning.pan@fudan.edu.cn

[#]Linyu Yan and Weiwei Jin contributed equally to this work.

This file includes:

Supplementary Figures
Supplementary Tables

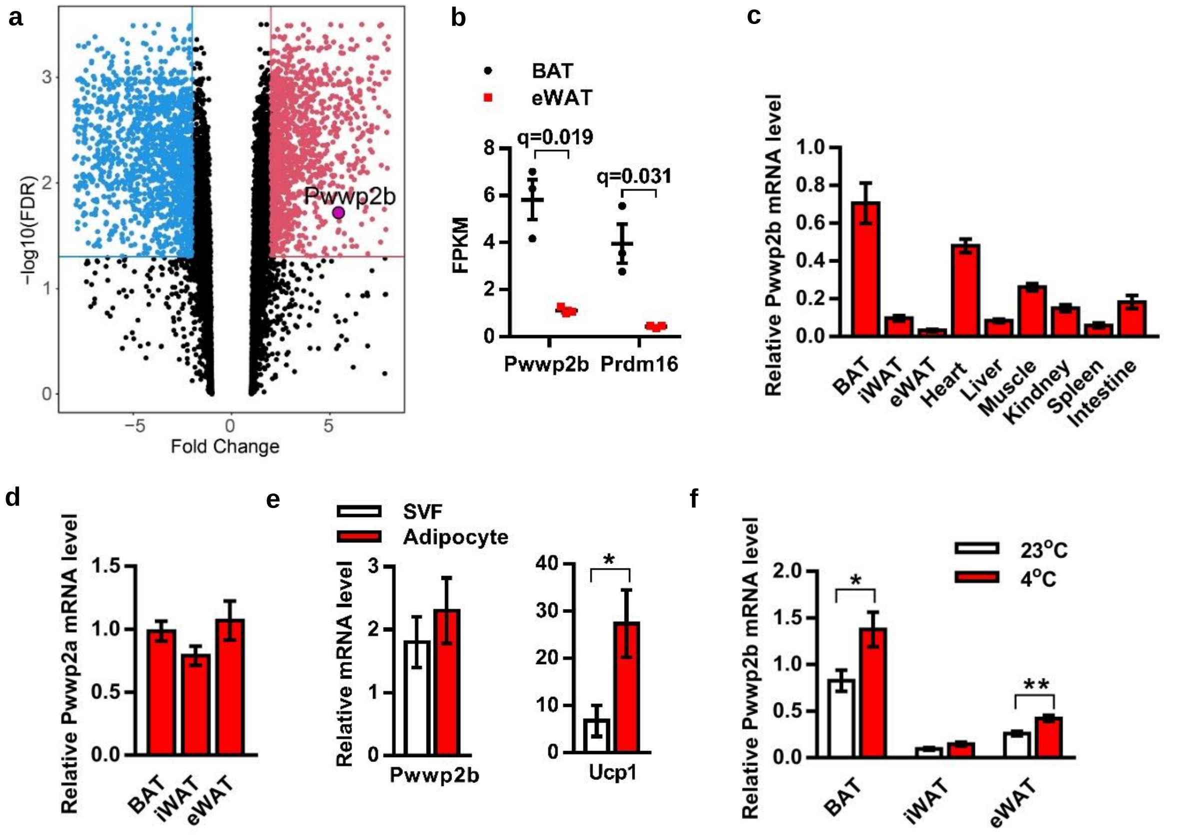

Figure S1. ***Pwwp2b* is a BAT-enriched gene relative to WAT.**

a) Volcano plots of RNA-seq (GEO: GSE56367). Horizontal axis represents relative fold of gene levels comparing BAT to eWAT. n=3.

b) *Pwwp2b* and *Prdm16* reads in RNA-seq. *Prdm16* is shown as a positive control. FPKM: fragments per kilobase of exon per million reads. FDR q value is shown.

c) RT-qPCR analysis of *Pwwp2b* mRNA level in C57BL/6 mouse tissues (n=4).

d) RT-qPCR analysis of *Pwwp2a* mRNA level in BAT, iWAT and eWAT (n=4).

e) *Pwwp2*b (left) and *Ucp1* (right) mRNA levels in SVF and mature adipocytes isolated from brown fat tissues. n=3.

f) *Pwwp2b* mRNA level in fat tissues after acute cold exposure for 6 hours (n=5).

*P<0.05 by unpaired two-tailed Student's t-test. The data shown are mean±SEM.

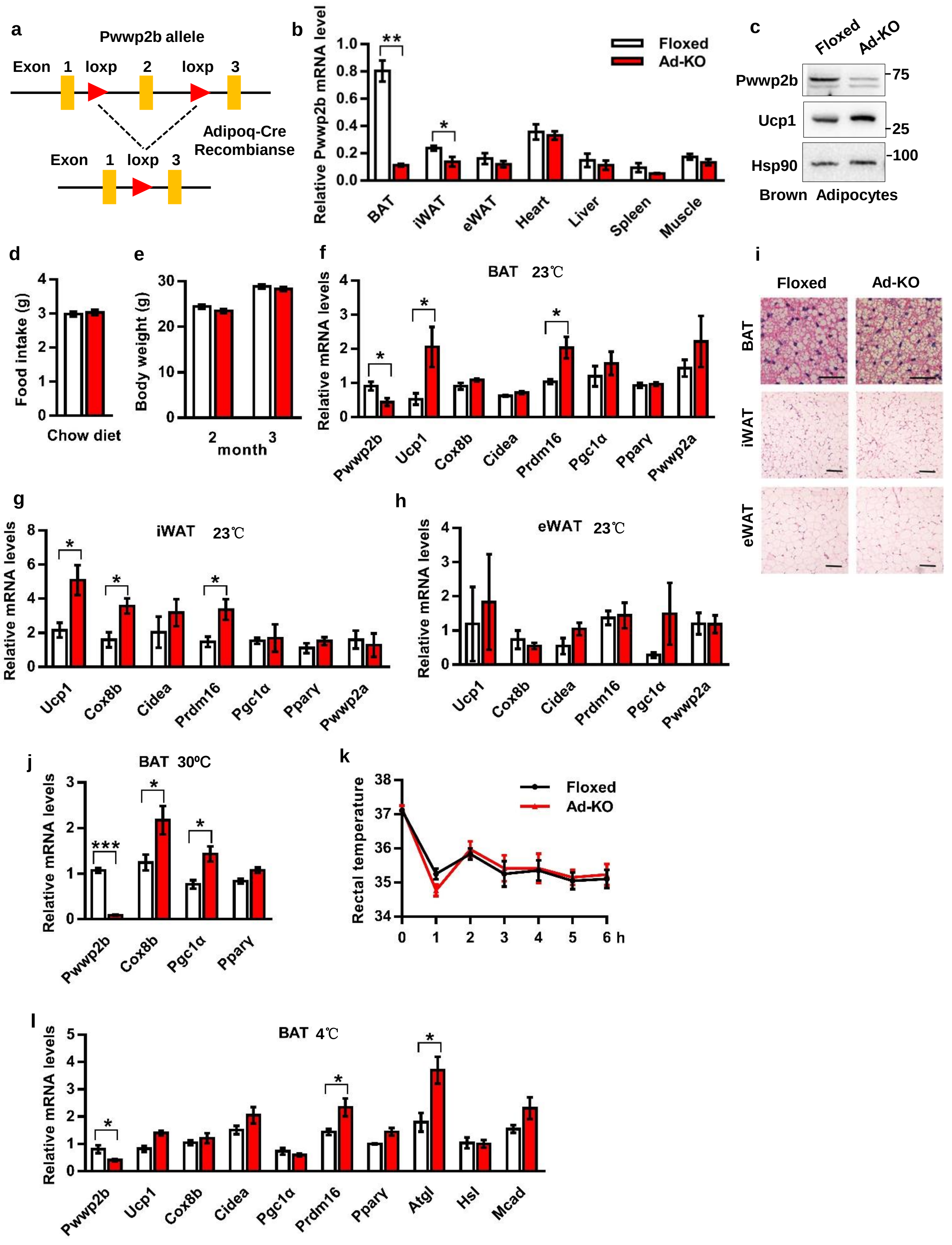
a
Pwwp2b allele
Exon 1 loxp 2 loxp 3
Adipoq-Cre Recombianse
Exon 1 loxp 3
b
Relative Pwwp2b mRNA level
Floxed
Ad-KO
BAT iWAT eWAT Heart Liver Spleen Muscle
c
Floxed Ad-KO
Pwwp2b
Ucp1
Hsp90
75
25
100
Brown Adipocytes
d
Food intake (g)
Chow diet
e
Body weight (g)
2 3 month
f
BAT 23℃
Relative mRNA levels
Pwwp2b Ucp1 Cox8b Cidea Prdm16 Pgc1α Pparγ Pwwp2a
i
Floxed Ad-KO
BAT
iWAT
eWAT
g
iWAT 23℃
Relative mRNA levels
Ucp1 Cox8b Cidea Prdm16 Pgc1α Pparγ Pwwp2a
h
eWAT 23℃
Relative mRNA levels
Ucp1 Cox8b Cidea Prdm16 Pgc1α Pwwp2a
j
BAT 30ºC
Relative mRNA levels
Pwwp2b Cox8b Pgc1α Pparγ
k
Rectal temperature
Floxed
Ad-KO
0 1 2 3 4 5 6 h
l
BAT 4℃
Relative mRNA levels
Pwwp2b Ucp1 Cox8b Cidea Pgc1α Prdm16 Pparγ Atgl Hsl Mcad

Figure S2. *Pwwp2b* deletion increases thermogenic gene expression in adipose tissues.

a) Strategy to generate adipose tissue *Pwwp2b*-knockout mice.

b) Pwwp2b mRNA level in Floxed and *Pwwp2b*-knockout mice (n=4).

c) PWWP2B and UCP1 protein levels in mature brown adipocytes isolated from Floxed and *Pwwp2b*-knockout mice. Each sample was generated by pooling adipocytes from 3 mice together.

d) Chow diet intake of *Pwwp2b* Floxed and knockout male mice (n=8)..

e) Body weight of *Pwwp2b* Floxed and knockout male mice fed on a chow diet at the age of 2 and 3 months (n=5-7).

f-h) Gene expression in BAT (f), iWAT (g) and eWAT (h) of *Pwwp2b* Floxed and knockout mice. n=4-5, 9-week-old male mice.

i) Hematoxylin and Eosin (H&E) Staining of BAT (scale bar=20µm), iWAT and eWAT (scale bar=50µm) in mice fed a chow diet.

j) Gene expression of BAT from male mice raising at 30 ℃ for 2 weeks (n=5).

k) Rectal temperature of male mice during acute cold exposure to 4 ℃ (n=5).

l) Gene expression of BAT from male mice after 4 ℃ exposure for 7 hours (n=5).

*P<0.05, **P<0.01, ***P<0.001 by unpaired two-tailed Student’s t-test. The data shown are mean±SEM.

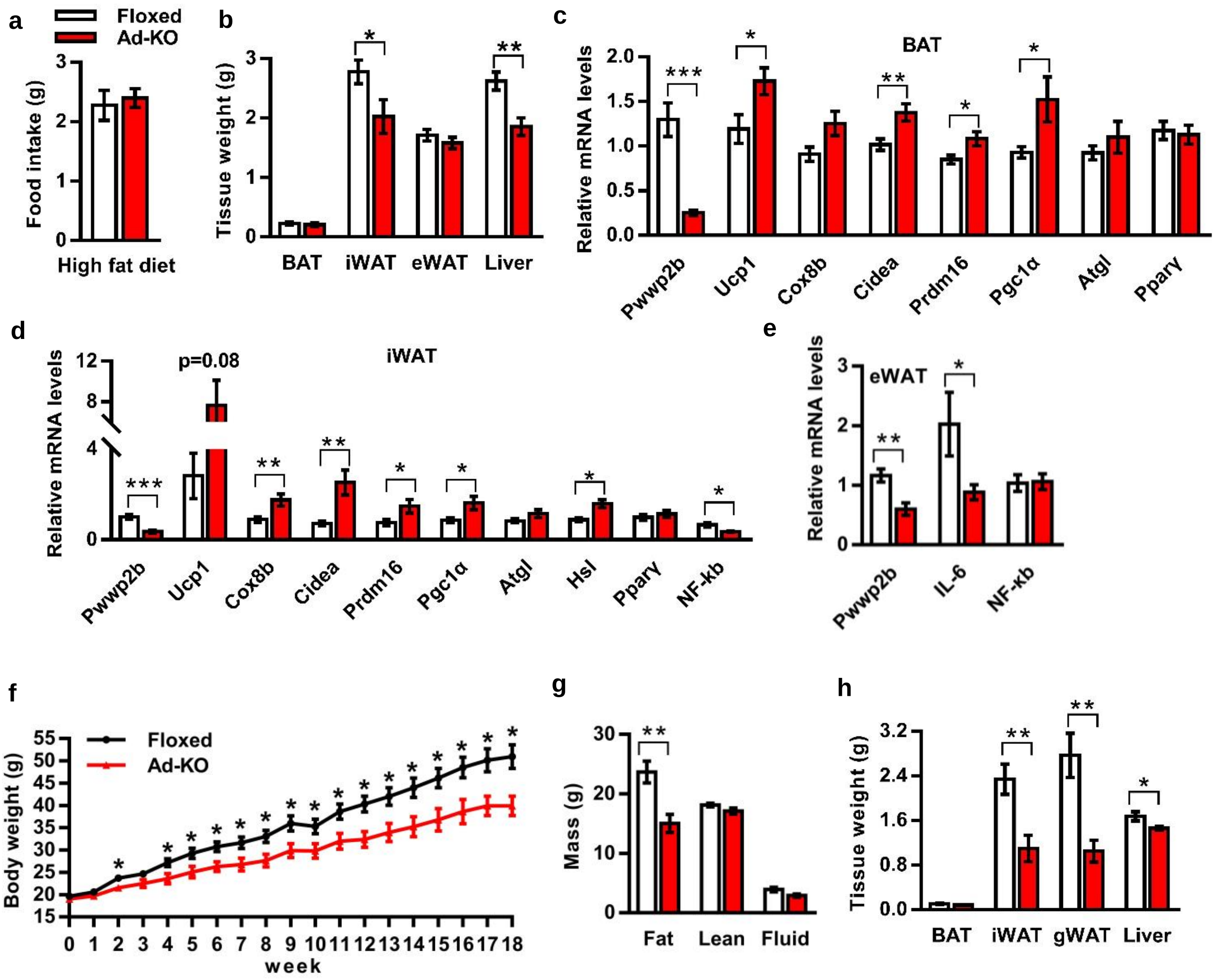

Figure S3. ***Pwwp2b*** **knockout mice are more resistance to HFD-induced obesity.**

a) Food intake of high-fat-diet. n=7.

b) Fat and liver weights of mice after a HFD-feeding for 11 weeks. n=10-11, male mice.

c-e) Gene expression in BAT (c), iWAT (d) and eWAT (e) of mice in (b). n=10-11, male mice.

f) Female *Pwwp2b* Floxed and knockout mice were fed a HFD for 18 weeks. Body weight was measure every week. n=5-6. *P<0.05 versus the control (0 week).

g) Body composition of mice in (f). n=5-6.

h) The weight of BATs, iWATs, gonadal WATs (gWAT) and livers in mice of (f). n=5-6.

*P<0.05, **P<0.01, ***P<0.001 by unpaired two-tailed Student's t-test. The data shown are mean±SEM.

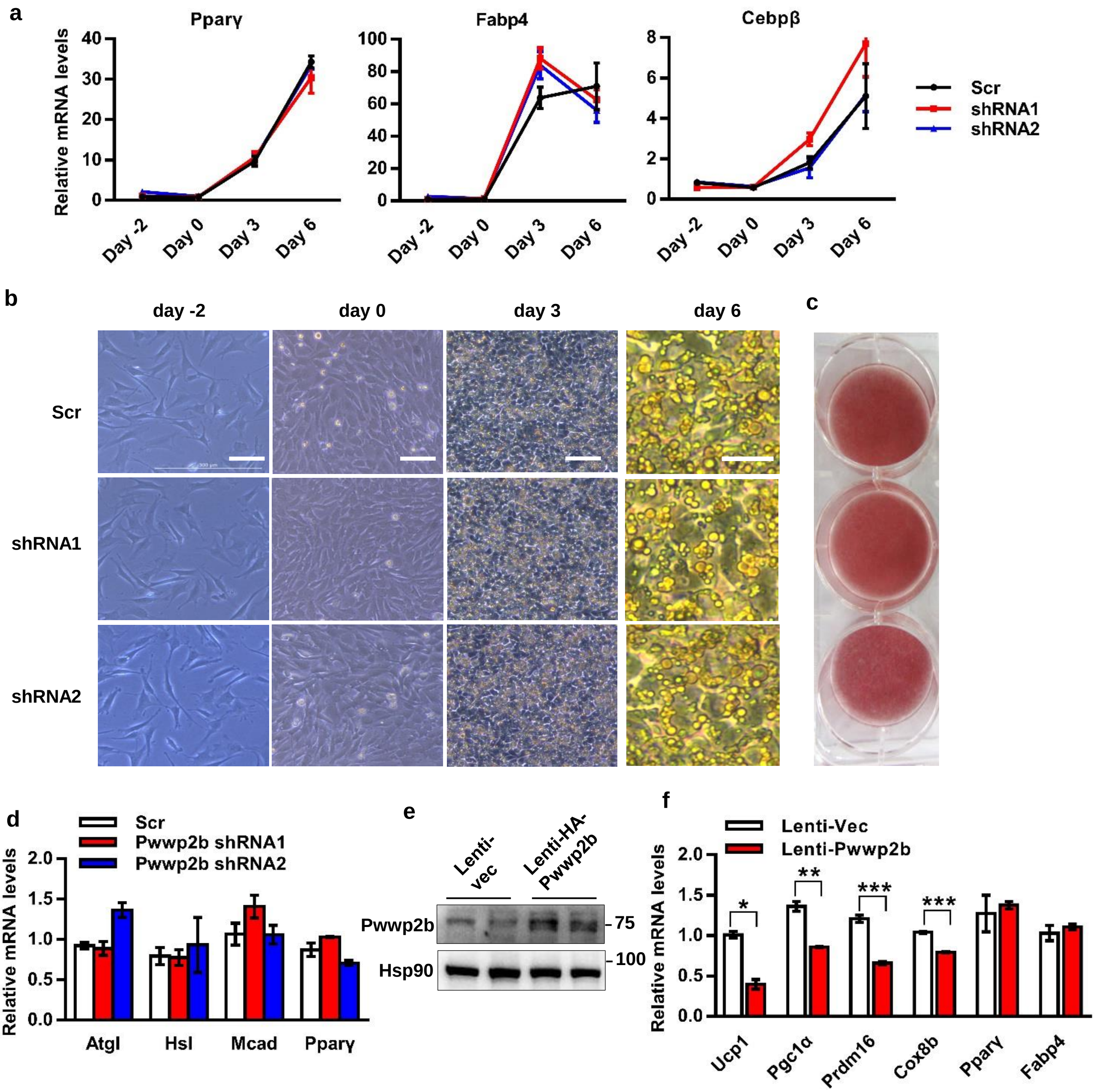
a
Pparγ
Fabp4
Cebpβ
Relative mRNA levels
Day -2
Day 0
Day 3
Day 6
Scr
shRNA1
shRNA2
b
day -2
day 0
day 3
day 6
Scr
shRNA1
shRNA2
c
d
Scr
Pwwp2b shRNA1
Pwwp2b shRNA2
Relative mRNA levels
Atgl
Hsl
Mcad
Pparγ
e
Lenti-vec
Lenti-HA-Pwwp2b
Pwwp2b
Hsp90
75
100
f
Lenti-Vec
Lenti-Pwwp2b
Relative mRNA levels
Ucp1
Pgc1α
Prdm16
Cox8b
Pparγ
Fabp4

Figure S4. PWWP2B suppressing thermogenic program is adipocyte-autonomous.

a) Lentiviral shRNAs against *Pwwp2b* or scrambled control were transduced to brown preadipocytes following puromycin selection. Then cells were induced to mature adipocytes. The expression of adipogenic genes was analyzed by QPCR. n=3.

b) Representative images of cells in (a) during differentiation taken by a phase-contrast microscope. Scale bar in day -2, 0 and 3 is 100μm; scale bar in day 6 is 50μm.

c) *Pwwp2b* knockdown cells generated as in (a) were stained by Oil red O on differentiation day 6.

d) Gene expression levels in *Pwwp2b* knockdown cells generated as in (a) detected by QPCR. n=3.

e) The PWWP2B level in lentiviral *Pwwp2b*-transduced adipocytes. The lentivirus bearing *Pwwp2b* infected brown preadipocytes. Cells were induced to mature adipocytes and PWWP2B protein level was analyzed on day 6.

f) Gene expression analysis in adipocytes in (e) was performed on day 6. n=3.

$^*P<0.05$, $^{**}P<0.01$, $^{***}P<0.001$ by unpaired two-tailed Student's t-test. The data shown are mean±SEM.

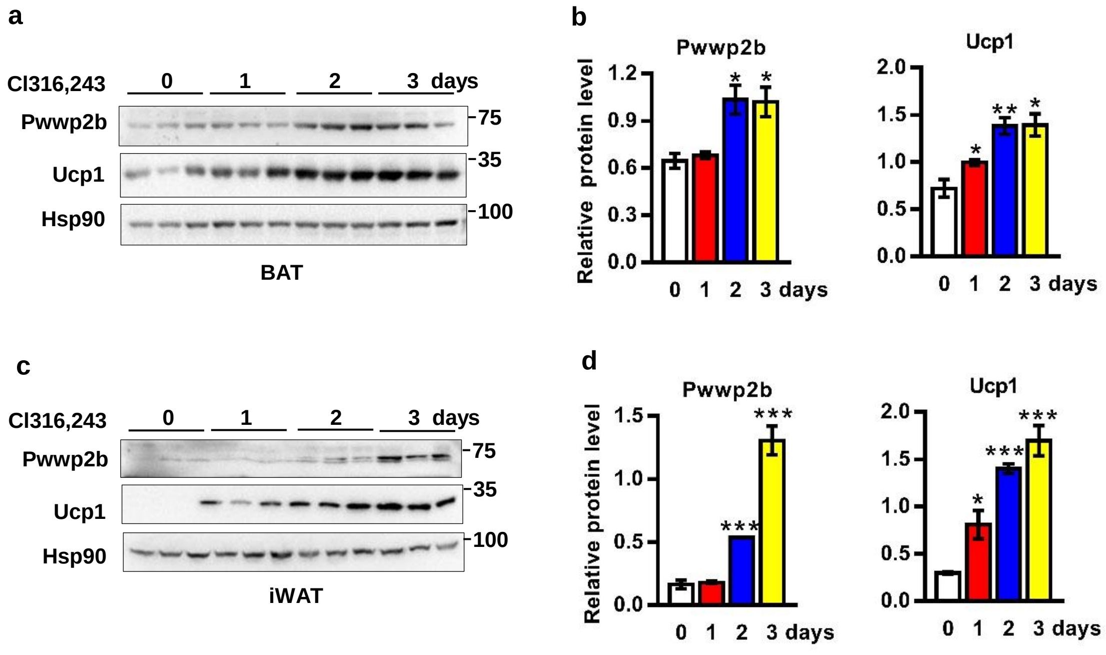

Figure S5. PWWP2B induction by Cl316,243 lags behind UCP1 induction.

a) C57BL/6 mice were injected Cl316,243 (0.5ug g^{-1} body weight) for indicated time. PWWP2B and UCP1 levels were analyzed in BATs by western blot. n=3.

b) Quantification of PWWP2B and UCP1 protein levels in (a). n=3.

c) PWWP2B and UCP1 levels in inguinal WATs from mice in (a). n=3.

d) Quantification of PWWP2B and UCP1 protein levels in (c). n=3.

*P<0.05, **P<0.01, ***P<0.001 versus the control (0 day) by unpaired two-tailed Student's t-test. The data shown are mean±SEM.

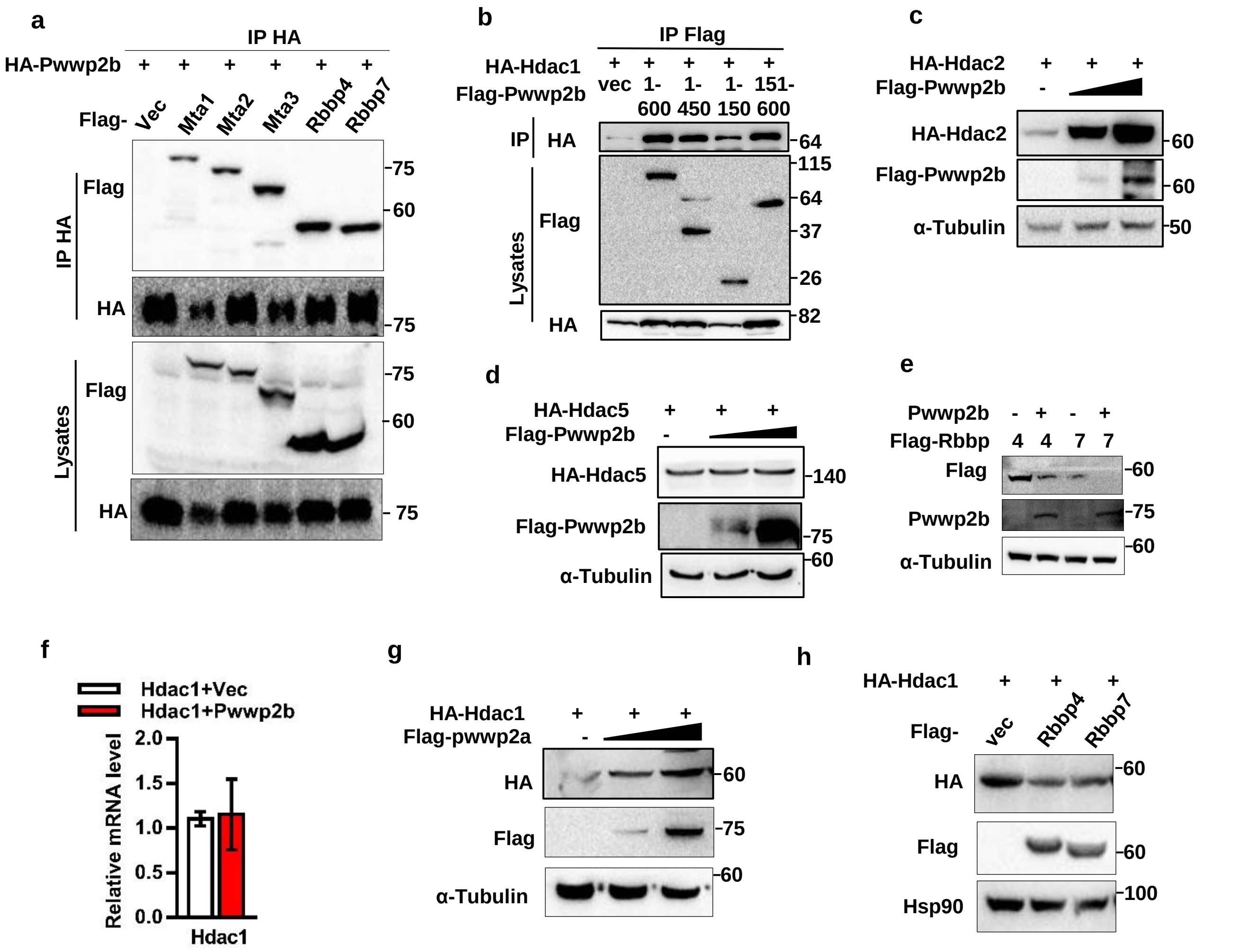
a
IP HA
HA-Pwwp2b + + + + + +
Flag- Vec Mta1 Mta2 Mta3 Rbbp4 Rbbp7
IP HA
Flag
HA
Lysates
Flag
HA
75
60
b
IP Flag
HA-Hdac1 + + + + +
Flag-Pwwp2b vec 1-600 1-450 1-150 151-600
IP HA
Lysates
Flag
HA
64
115
37
26
82
c
HA-Hdac2 + + +
Flag-Pwwp2b -
HA-Hdac2
Flag-Pwwp2b
α-Tubulin
50
d
HA-Hdac5 + + +
Flag-Pwwp2b -
HA-Hdac5
Flag-Pwwp2b
α-Tubulin
140
e
Pwwp2b - + - +
Flag-Rbbp 4 4 7 7
Flag
Pwwp2b
α-Tubulin
f
Hdac1+Vec
Hdac1+Pwwp2b
Relative mRNA level
2.0
1.5
1.0
0.5
0.0
Hdac1
g
HA-Hdac1 + + +
Flag-pwwp2a -
HA
Flag
α-Tubulin
h
HA-Hdac1 + + +
Flag- vec Rbbp4 Rbbp7
HA
Flag
Hsp90
100

Figure S6. PWWP2B is a component of the NuRD subcomplex.

a) Plasmids as indicated were transfected to HEK293T cells by Lipofectamin 2000 and Immunoprecipitation assay was performed 48 h after transfection.

b) Plasmids as indicated were transfected to HEK293T cells. Immunoprecipitation assay to map the region on PWWP2B mediating interaction with HDAC1.

c) *Pwwp2b* and *Hdac2* plasmids were transfected to HEK293T cells. Western blot was performed 48 h after transfection.

d) *Pwwp2b* and *Hdac5* plasmids were transfected to HEK293T cells followed by western blot 48 h after transfection.

e) *Hdac1* and *Rbbp4* or *Rbbp7* plasmids were co-transfected to HEK293T cells. Western blot analysis was performed 48 hours after transfection.

f) *Hdac1* plasmid was co-transfected into HEK293T cells together with *Pwwp2b* or empty vector plasmids. QPCR was performed 48 h after transfection. n=3. Unpaired two-tailed Student's t-test. The data shown are mean±SEM.

g) PWWP2A stabilizes HDAC1 when co-expressed in HEK293T cells. *Pwwp2a* and *Hdac1* plasmids were transfected to HEK293T cells. Western blot was performed 48 h after transfection.

h) The presence of RBBP4 or RBBP7 does not increase HDAC1 protein level in HEK293T cells. *Rbbp4* or *Rbbp7* plasmids were co-transfected with *Hdac1* plasmids to HEK293T cells. Western blot analysis was performed 48 h after transfection.

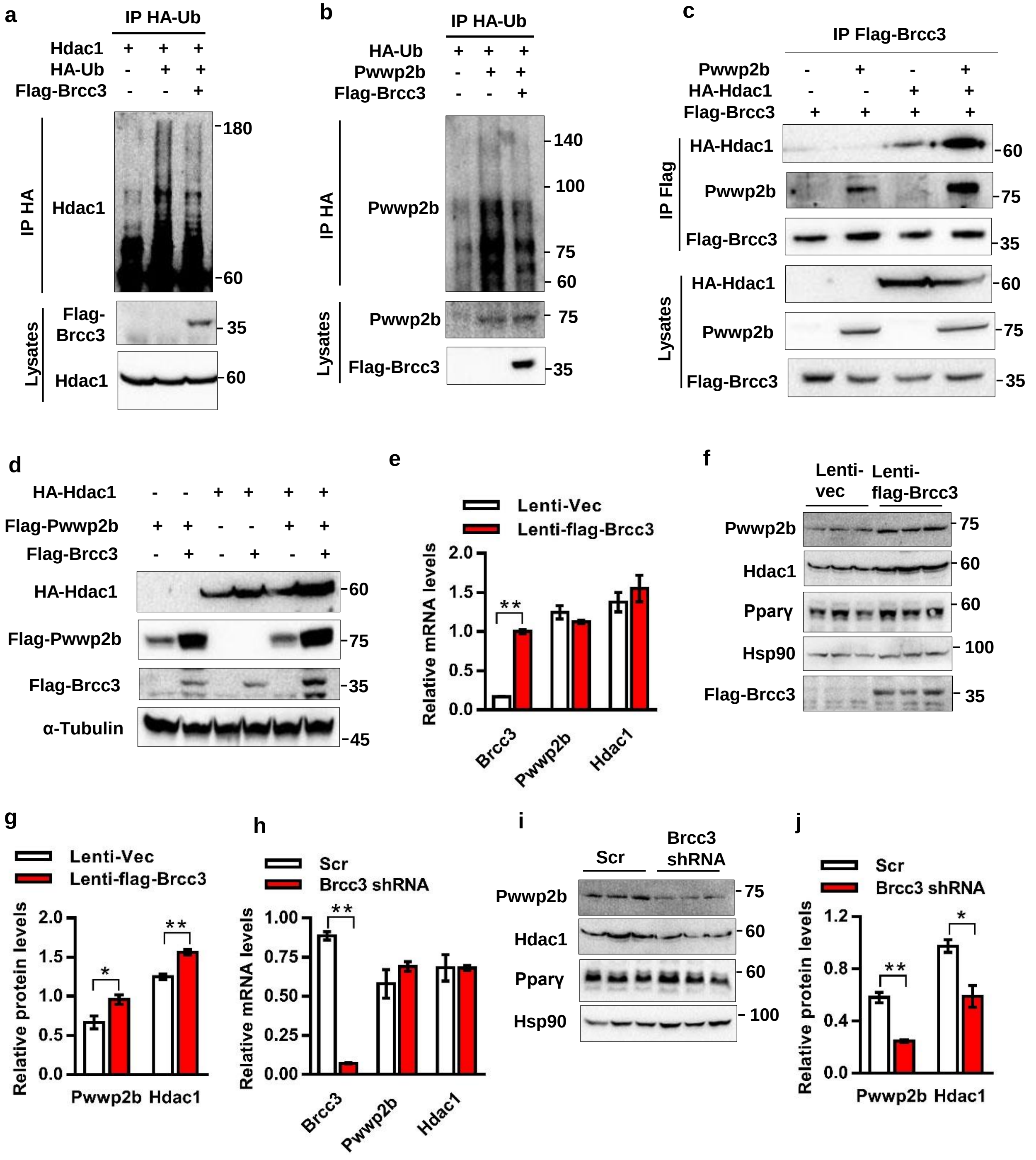
a
IP HA-Ub
Hdac1
HA-Ub
Flag-Brcc3
IP HA
Hdac1
180
60
Lysates
Flag-Brcc3
35
Hdac1
60
b
IP HA-Ub
HA-Ub
Pwwp2b
Flag-Brcc3
IP HA
Pwwp2b
140
100
75
60
Lysates
Pwwp2b
75
Flag-Brcc3
35
c
IP Flag-Brcc3
Pwwp2b
HA-Hdac1
Flag-Brcc3
IP Flag
HA-Hdac1
60
Pwwp2b
75
Flag-Brcc3
35
Lysates
HA-Hdac1
60
Pwwp2b
75
Flag-Brcc3
35
d
HA-Hdac1
Flag-Pwwp2b
Flag-Brcc3
HA-Hdac1
60
Flag-Pwwp2b
75
Flag-Brcc3
35
α-Tubulin
45
e
Lenti-Vec
Lenti-flag-Brcc3
Relative mRNA levels
2.0
1.5
1.0
0.5
0.0
**
Brcc3
Pwwp2b
Hdac1
f
Lenti-vec
Lenti-flag-Brcc3
Pwwp2b
75
Hdac1
60
Pparγ
60
Hsp90
100
Flag-Brcc3
35
g
Lenti-Vec
Lenti-flag-Brcc3
Relative protein levels
2.0
1.5
1.0
0.5
0.0
*
**
Pwwp2b
Hdac1
h
Scr
Brcc3 shRNA
Relative mRNA levels
1.00
0.75
0.50
0.25
0.00
**
Brcc3
Pwwp2b
Hdac1
i
Scr
Brcc3 shRNA
Pwwp2b
75
Hdac1
60
Pparγ
60
Hsp90
100
j
Scr
Brcc3 shRNA
Relative protein levels
1.2
0.8
0.4
0.0
**
*
Pwwp2b
Hdac1

Figure S7. BRCC3 enhances PWWP2B interacting with HDAC1.

a,b) BRCC3 deubiquitinates HDAC1 (a) and PWWP2B (b). Plasmids as indicated were transfected to HEK293T cells. Thirty-six hours after transfection MG132 (5μM) was supplemented in the culture media for another 12 h following immunoprecipitation with HA antibody.

c) PWWP2B facilitates BRCC3 interaction with HDAC1. Immunoprecipitation assay was performed 48 h after indicated plasmids were transfected to HEK293T cells.

d) PWWP2B enhances BRCC3 stabilizing effect on the HDAC1 protein. Plasmids as indicated were transfected to HEK293T cells. Western blot was performed 48 h after transfection.

e) Lentiviral *Brcc3* infected brown preadipocytes. Gene mRNA levels were analyzed in mature adipocytes (day 6) by QPCR. n=3.

f) Western blot was performed in cells generated as in (e). Ectopic expression of *Brcc3* increases PWWP2B and HDAC1 protein levels in mature brown fat cells.

g) Quantification of PWWP2B and HDAC1 protein levels in (f). n=3.

h) Brcc3 was knocked down by shRNAs in brown preadipocytes. Gene mRNA levels were analyzed in mature adipocytes (day 6) by QPCR. n=3.

i) Western blot was performed in cells generated as in (h). Knockdown of *Brcc3* decreases PWWP2B and HDAC1 protein levels in mature brown fat cells.

j) Quantification of PWWP2B and HDAC1 protein levels in (i). n=3.

*P<0.05, **P<0.01, ***P<0.001 by unpaired two-tailed Student's t-test. The data shown are mean±SEM.

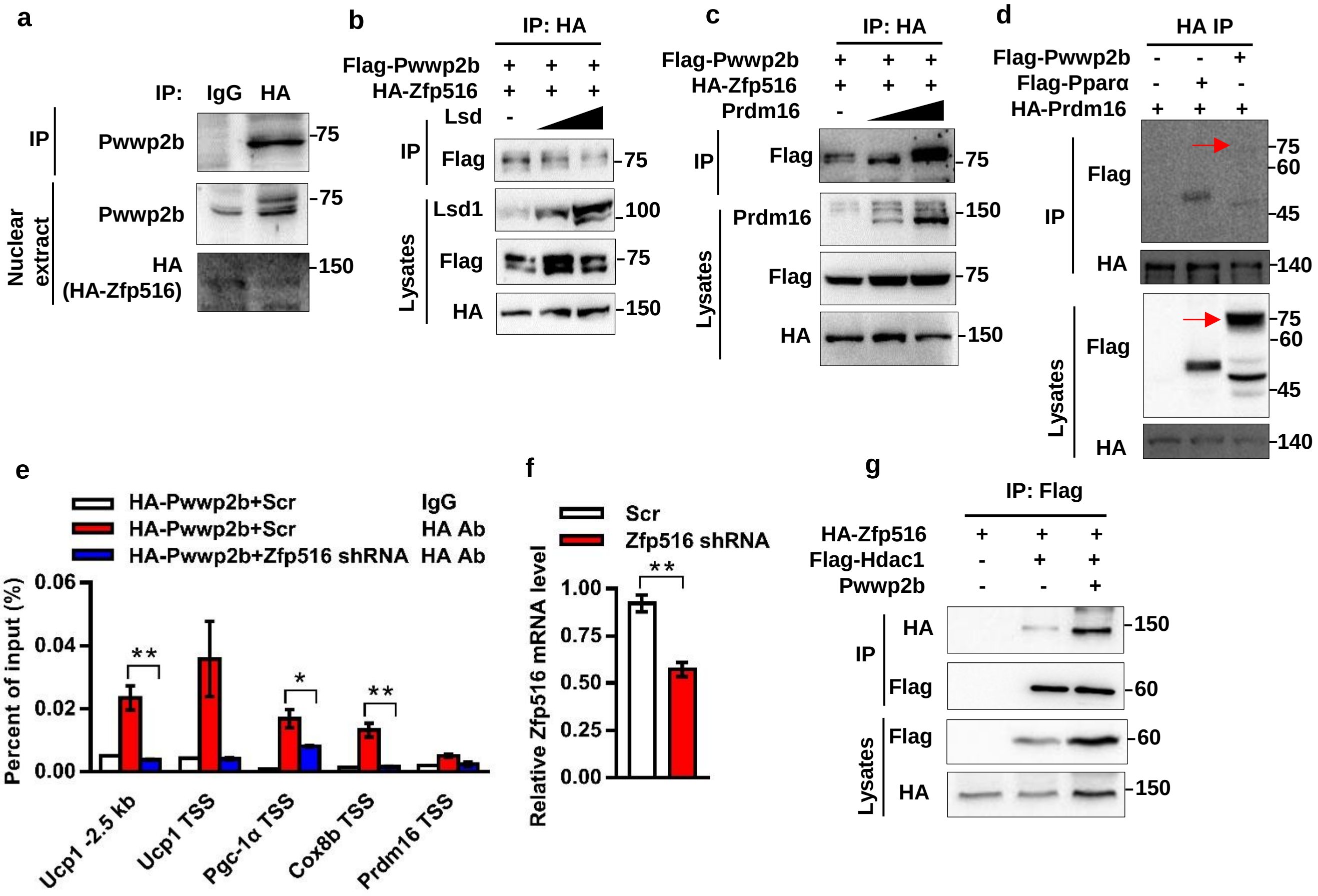
a
IP: IgG HA
IP Pwwp2b
Nuclear extract Pwwp2b
HA (HA-Zfp516)
75
75
150
b
IP: HA
Flag-Pwwp2b + + +
HA-Zfp516 + + +
Lsd -
IP Flag
Lysates Lsd1
Flag
HA
75
100
75
150
c
IP: HA
Flag-Pwwp2b + + +
HA-Zfp516 + + +
Prdm16 -
IP Flag
Lysates Prdm16
Flag
HA
75
150
75
150
d
HA IP
Flag-Pwwp2b - - +
Flag-Pparα - + -
HA-Prdm16 + + +
IP Flag
HA
Lysates Flag
HA
75
60
45
140
75
60
45
140
e
HA-Pwwp2b+Scr IgG
HA-Pwwp2b+Scr HA Ab
HA-Pwwp2b+Zfp516 shRNA HA Ab
Percent of input (%)
0.06
0.04
0.02
0.00
Ucp1 -2.5 kb
Ucp1 TSS
Pgc-1α TSS
Cox8b TSS
Prdm16 TSS
f
Scr
Zfp516 shRNA
Relative Zfp516 mRNA level
1.00
0.75
0.50
0.25
0.00
g
IP: Flag
HA-Zfp516 + + +
Flag-Hdac1 - + +
Pwwp2b - - +
IP HA
Flag
Lysates Flag
HA
150
60
60
150

Figure S8. ZFP516 recruits PWWP2B to regulate thermogenic program..

a) Overexpressed ZFP516 interacts with endogenous PWWP2B in brown fat cells. ZFP516 was overexpressed by lentiviral transduction to preadipocytes. Immunoprecipitation assay was performed in mature adipocytes (day 6).

b) PWWP2B competes LSD1 to associate with ZFP516. Plasmids were transfected to HEK293T cells following the immunoprecipitation assay.

c) PRDM16 promotes PWWP2B interacting with ZFP516. Plasmids were transfected to HEK293T cells following the immunoprecipitation assay.

d) The interaction between PWWP2B and PRDM16 can not be detected. The plasmid bearing *Prdm16* was co-transfected with a *Pwwp2b* or *Pparα* plasmid to HEK293T cells. The interaction between PPARα and PRDM16 was used as a positive control. The red arrows point to PWWP2B in western blot.

e) Knocking down *Zfp516* abolished PWWP2B association with gene promoters. Lentivirus bearing HA-*Pwwp2b* infected brown preadipocyte following standard induction to mature adipocytes. Lentiviral *Zfp516* shRNA was used to transduce adipocytes on differentiation day 2 and 4. ChIP was performed on day 6 with HA antibody or control IgG. ChIP was repeated for 3 times and representative data were shown. n=3.

f) The *Zfp516* knockdown efficiency in cells of (e). n=3.

g) PWWP2B enhances the interaction between ZFP516 and HDAC1. Plasmids were transfected to HEK293T cells. Immunoprecipitation was performed 48 h after transfection.

*$P<0.05$, **$P<0.01$ by unpaired two-tailed Student's t-test. The data shown are mean±SEM.

Table S1 Proteins identified by LC-MS/MS

Protein ID	Protein Name	
E9Q9M8	PWWP domain-containing 2B	Pwwp2b
A2AFI9	Histone-binding protein RBBP7	Rbbp7
Q3TZP3	Metastasis associated 1 family member 2	Mta2
Q58E49	Histone deacetylase 1	Hdac1
A0A0R4J008	Histone deacetylase 2	Hdac2
A2AFI9	Histone-binding protein RBBP4	Rbbp4
A4FTZ3	Metastasis associated 1 family member 3	Mta3
Q9CQ75	NADH dehydrogenase [ubiquinone] 1 alpha subcomplex subunit 2	Ndufa2
Z4YJV4	2-oxoglutarate dehydrogenase, mitochondrial	Ogdh
Q2YDW0	MICOS complex subunit Mic60	Immt
Q8R5L1	Complement component 1 Q subcomponent-binding protein, mitochondrial	C1qbp
Q8BH95	Enoyl-CoA hydratase, mitochondrial	Echs1
Q54AA6	Diacylglycerol O-acyltransferase 1	Dgat1
Q3TLH6	Fatty acid-binding protein, epidermal	Fabp5
Q3UAX2	Lipoprotein lipase	Lpl
X5J5H3	Zinc finger protein 536	Zfp536
B2RWD2	Zinc finger protein 516	Zfp516
Q3TZP3	Y-box-binding protein 1	Ybx1
A3KGA8	BRCA1/BRCA2-containing complex subunit 3	Brcc3
Q9D023	Mitochondrial pyruvate carrier 2	Mpc2
Q9WVL3	Solute carrier family 12 member 7	Slc12a2
Q5U4C5	Importin-4	Ipo4
Q543M7	Importin subunit alpha	Kpna3
P13541	Myosin-9	Myh9
Q3UF82	Mitogen-activated protein kinase	Mapk1
Q8C1Y3	Histone H1.0	H1f0
Q5RJV3	RNA-binding protein 3	Rbm3

Table S2

Primers used in this study

**shRNA sequences**	
Pwwp2b shRNA 1	5'-GACAGCCTGGATGAATTAA-3'
Pwwp2b shRNA 2	5'-AGAAGACAGCGCTGTCATAG-3'
Brcc3 shRNA	5'-CCAACAGCATTTGCAGGAATT-3'
Zfp516 shRNA	5'-CTTTACGGTGTATGGCGTTAT-3'
**ChIP-qPCR primers**	
Ucp1 chip PPRE	F: 5'-CTCCTCTACAGCGTCACAGAGG-3'
	R: 5'-AGTCTGAGGAAAGGGTTGA-3'
Ucp1 chip TSS	F: 5'-TGCCAAGTCCCACTAGCAG-3'
	R: 5'-ACCCGTTAAGCCCAGATTG-3'
Pgc1α chip TSS	F: 5'-CAAGCTTGCACAGGAGAAGG-3'
	R: 5'-CCAGCCCCTTACTGAGAGTG-3'
Prdm16 chip TSS	F: 5'-GCGACGAAGAGGATGATGA-3'
	R: 5'-TGTCAGTTTGGACACCTTCG-3'
Cox8b chip TSS	F: 5'-CAGTTACCAGCAGCCACCTT-3'
	R: 5'-CAAAGAGGCACCCTGAATGT-3'
Cidea chip TSS	F: 5'-CACGCACACCTGCTTCTCTA-3'
	R: 5'-GATGTTGGTGGCTCTTGTCA-3'
**real-time qPCR primers**	
Pwwp2b	F: 5'-GGAGCTCTTGACCCAGTTTG-3'
	R: 5'-CCCTGGGACAGAAGTCAGAA-3'
Pwwp2a	F: 5'-CTCAGCAGGTCTCAACAAATGGCA-3'
	R: 5'-CTGTCACATCCAAGAAACCAGCAG-3'
Ucp1	F: 5'-GGATTGGCCTCTACGACTCA-3'
	R: 5'-TGCCACACCTCCAGTCATTA-3'
Prdm16	F: 5'-CAGCACGGTGAAGCCATTC-3'
	R: 5'-GCGTGCATCCGCTTGTG-3'
Pgc-1α	F: 5'-AACCACACCCACAGGATCAGA-3'
	R: 5'-TCTTCGCTTTATTGCTCCATGA-3'
Cox8b	F: 5'-GAA CCA TGA AGC CAA CGA CT-3'
	R: 5'-GCG AAG TTC ACA GTG GTT CC-3'
Cidea	F: 5'-CGGGTAGTAAGTATGTCCCA-3'
	R: 5'-CAGCATAGGACATAAACCTCA-3'
Dio2	F: 5'-TTCCTGGCGCTCTATGACTC-3'
	R: 5'-AACCTGTTTGTAGGCATCTAGGA-3'
Pparγ	F: 5'-CAAGAATACCAAAGTGCGATCAA-3'
	R: 5'-GAGCTGGGTCTTTTCAGAATAATAAG-3'
Fabp4	F: 5'-GGCGTGACTTCCACAAGAGTTTA-3'
	R: 5'-GCCTCTTCCTTTGGCTCATG-3'
Cebpβ	F: 5'-CAAGCTGAGCGACGAGTACA-3'
	R: 5'-GACAGCTGCTCCACCTTCTT-3'
Agtl	F: 5'-CAACGCCACTCACATCTACG-3'
	R: 5'-CAATAATGTTGGCACCTGCTT-3'
Hsl	F: 5'-ACGCTACACAAAGGCTGCTT-3'
	R: 5'-TCTCGTTGCGTTTGTAGTGC-3'

Mcad	F: 5'-GCCAAGATCTATCAGATTTATGAAGGT-3'
	R: 5'-AGCTATGATCAGCCTCTGAATTTGT-3'
IL-6	F: 5'-GAGTTGTGCAATGGCAATTC-3'
	R: 5'-GGTACTCCAGAAGACCAGAG-3'
NF-κb	F: 5'-CTCATCTTTCCCTCAGAGCC-3'
	R: 5'-TCTCTCGCCAGGAATACTGC-3'
Hdac1(human)	F: 5'-GTTCTGTGGCAAGTGCTGT-3'
	R: 5'-TCAATGTACAGCACCCTCTG-3'
Hdac1(mouse)	F: 5'-AGTCTGTTACTACTACGACGGG-3'
	R: 5'-TGAGCAGCAAATTGTGAGTCAT-3'
Brcc3	F: 5'-GGCCTTCACATGTTGATGTTCG-3'
	R: 5'-GGCTTGTATGGATTGGAAGC-3'
Zfp516	F: 5'-CACAGAGGGACATCACTAAC-3'
	R: 5'-AGAATCTCGTGGACCGTCACA-3'

Table S3

Antibodies used in this study

Company	Antibody	Product code	Dilution ratio
Sigma	anti-Pwwp2b	SAB1104449	1:1500
	anti-Flag	SAB4301135	1:3000
Abcam	anti-Pwwp2b	ab110052	1:1000
	anti-Ucp1	ab10983	1:2000
	anti-Prdm16	ab106410	1:1000
	anti-Pparγ1/2	ab41928	1:1000
Proteintech	anti-Cidea	13170-1-AP	1:500
	anti-Hdac1	10197-1-AP	1:1000
	anti-Lsd1	20813-1-AP	1:2000
	anti-Hsp90	13171-1-AP	1:5000
	anti-α-Tubulin	66031-1-Ig	1:5000
	anti-Akt2	17609-1-AP	1:1000
Absin	anti-p-AKT	abs130891	1:1000
Santa cruz	anti-HA	sc-805X	1:3000
Smart lifesciences	anti-HA beads	SA068001	Immunoprecipitation
Smart lifesciences	anti-Flag beads	SA042001	Immunoprecipitation
Santa cruz	anti-HA	sc-805X	ChIP
Proteintech	anti-Hdac1	10197-1-AP	ChIP
Abcam	anti-acetyl H3K2	ab4729	ChIP
Santa cruz	anti-acetyl H4	sc-34263	ChIP
